# Supplementary material for: Activation and substrate specificity of the human P4-ATPase ATP8B1
Source: Nat Commun. 2023 Nov 18;14:7492. doi: 10.1038/s41467-023-42828-9 (PMC10657443; doi:10.1038/s41467-023-42828-9)

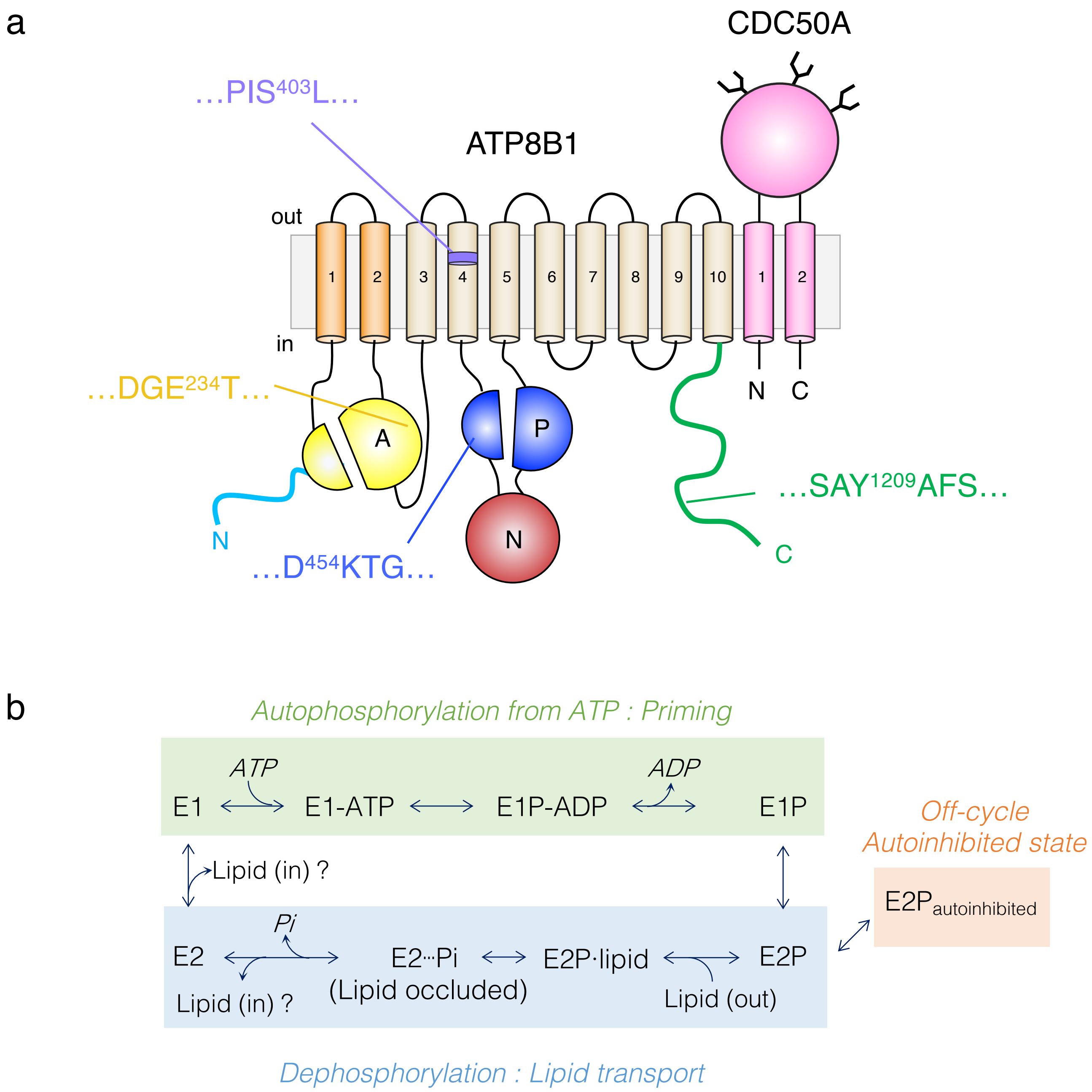

Supplementary Figure 1 – ATP8B1-CDC50A topology and Post-Albers P4-ATPase catalytic cycle.

a) The ATP8B1-CDC50A complex topology shows the Actuator domain (A), the Nucleotide binding domain (N), and the Phosphorylation domain (P) highlighted in yellow, red, and blue, respectively. The regulatory N- and C-terminal tails are shown in light blue and green, respectively. The P4-ATPase canonical motif of the phosphorylation site (DKTG, P domain), dephosphorylation loop (DGET, A domain), the transport site (PISL, TM4), and the autoinhibition signature motif (SAYAFS, C-terminal tail) are highlighted with ATP8B1 residue numbers. **b)** The Post-Albers P4-ATPase catalytic cycle includes the substrate-independent phosphorylation step from ATP (Priming) and the substrate-dependent dephosphorylation (transport). For autoinhibited P4-ATPases, an off-cycle E2P state prevents transport.

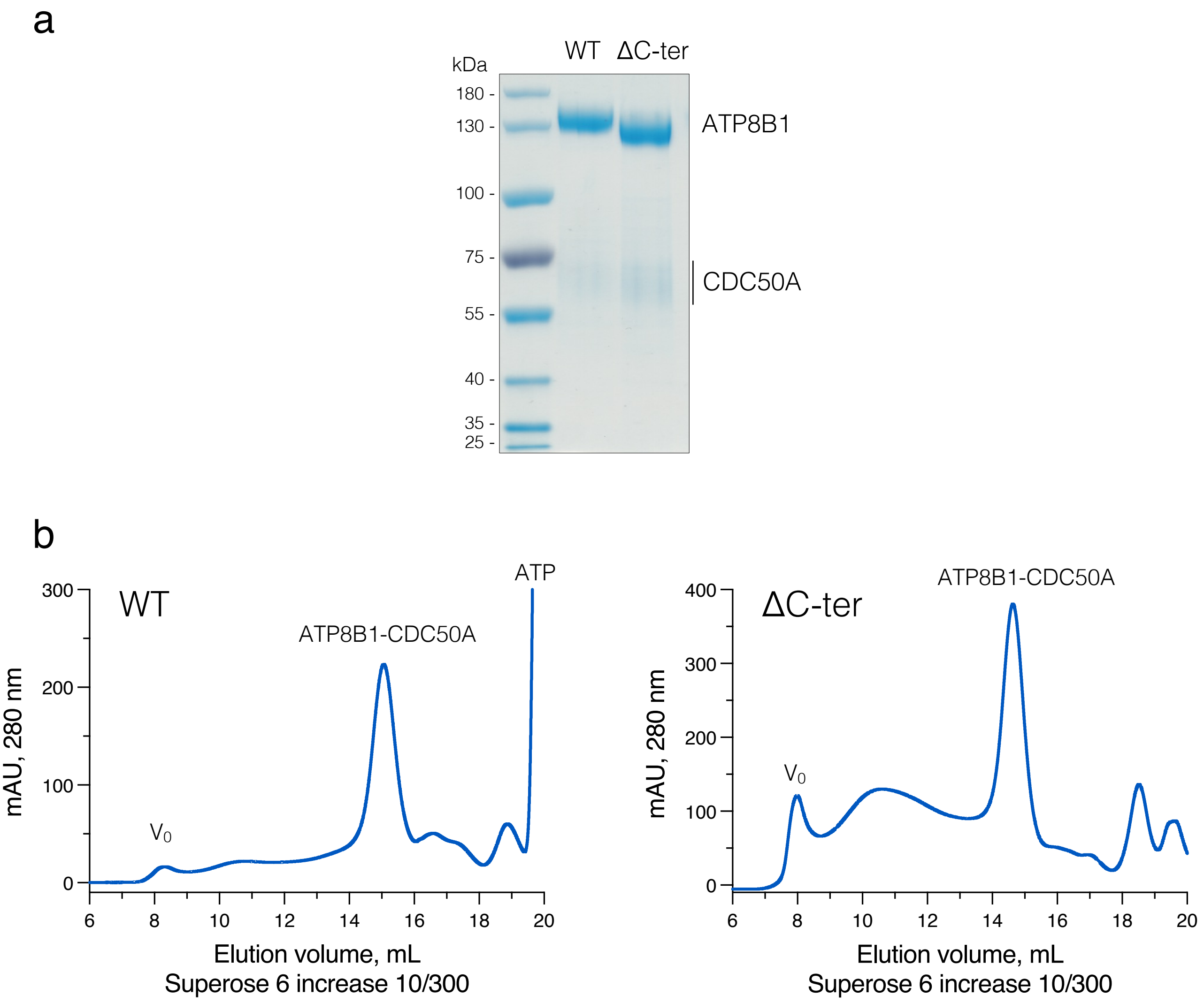

Supplementary Figure 2 – Biochemical characterization of the purified ATP8B1-CDC50A complex.

a) SDS-PAGE of the purified ATP8B1-CDC50A complexes (WT full-length and C-terminally truncated) used for cryo-EM and ATPase activity measurements in this study. **b)** Size exclusion chromatography (Superose 6 increase 10/300) elution profiles from the last purification step of the WT full-length and C-terminally truncated ATP8B1-CDC50A complexes.

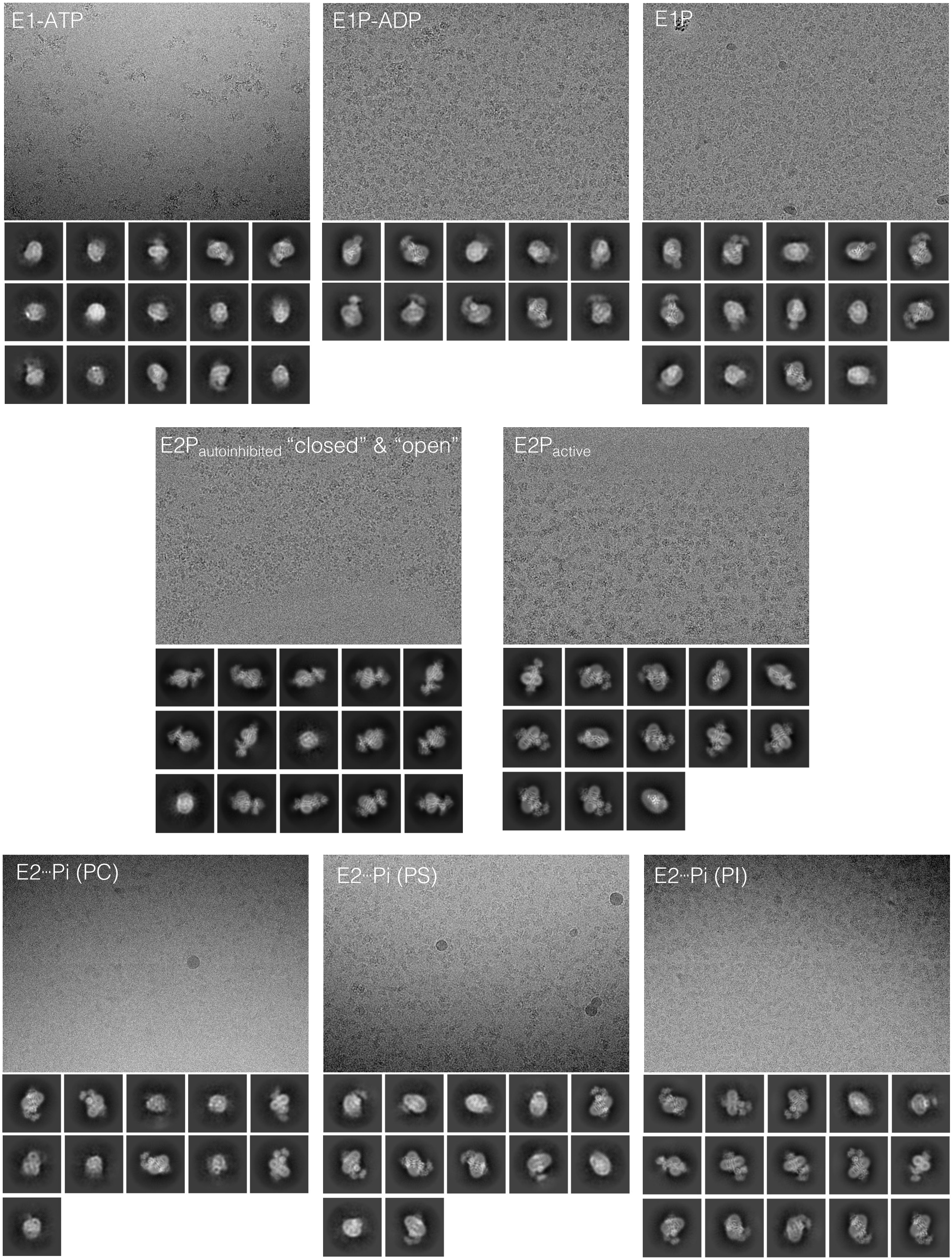

Supplementary Figure 3 – Representative micrographs and 2D classes of the particle stack used for final volume reconstruction of each data set.

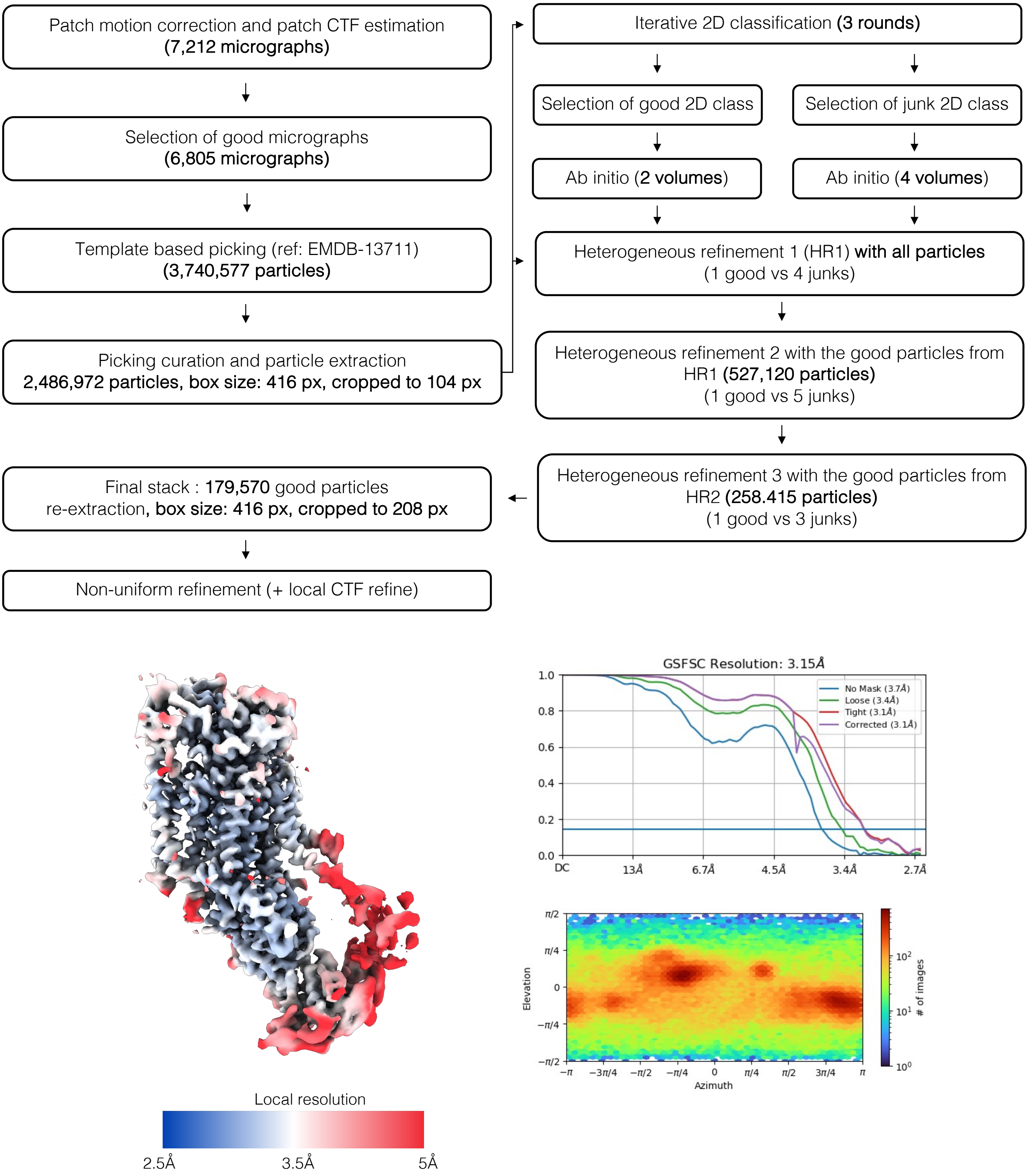

Supplementary Figure 4 – Cryo-EM data processing pipeline of ATP8B1-CDC50A (E1-ATP).

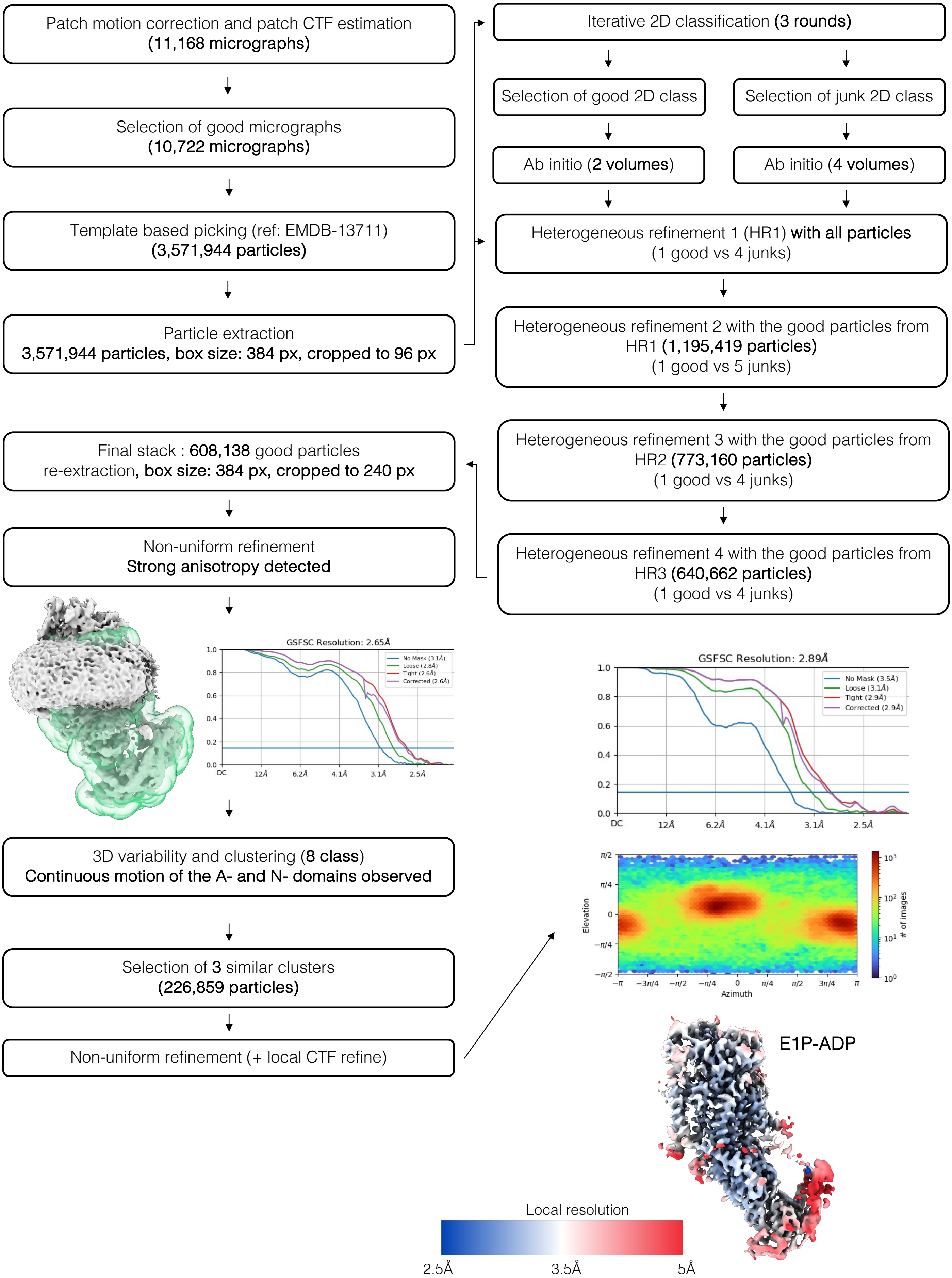

Supplementary Figure 5 – Cryo-EM data processing pipeline of ATP8B1-CDC50A (E1P-ADP).

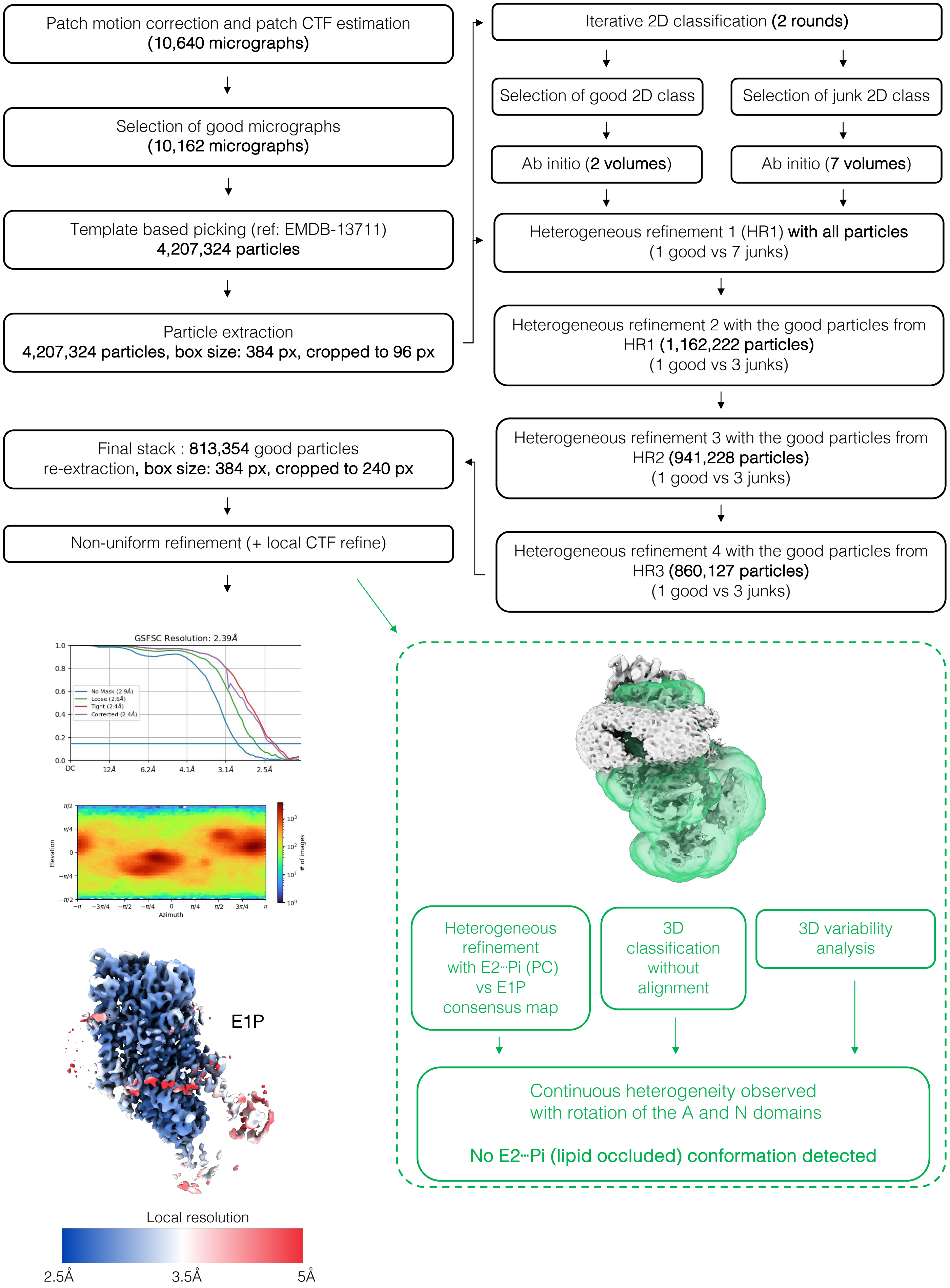

Supplementary Figure 6 – Cryo-EM data processing pipeline of ATP8B1-CDC50A (E1P).

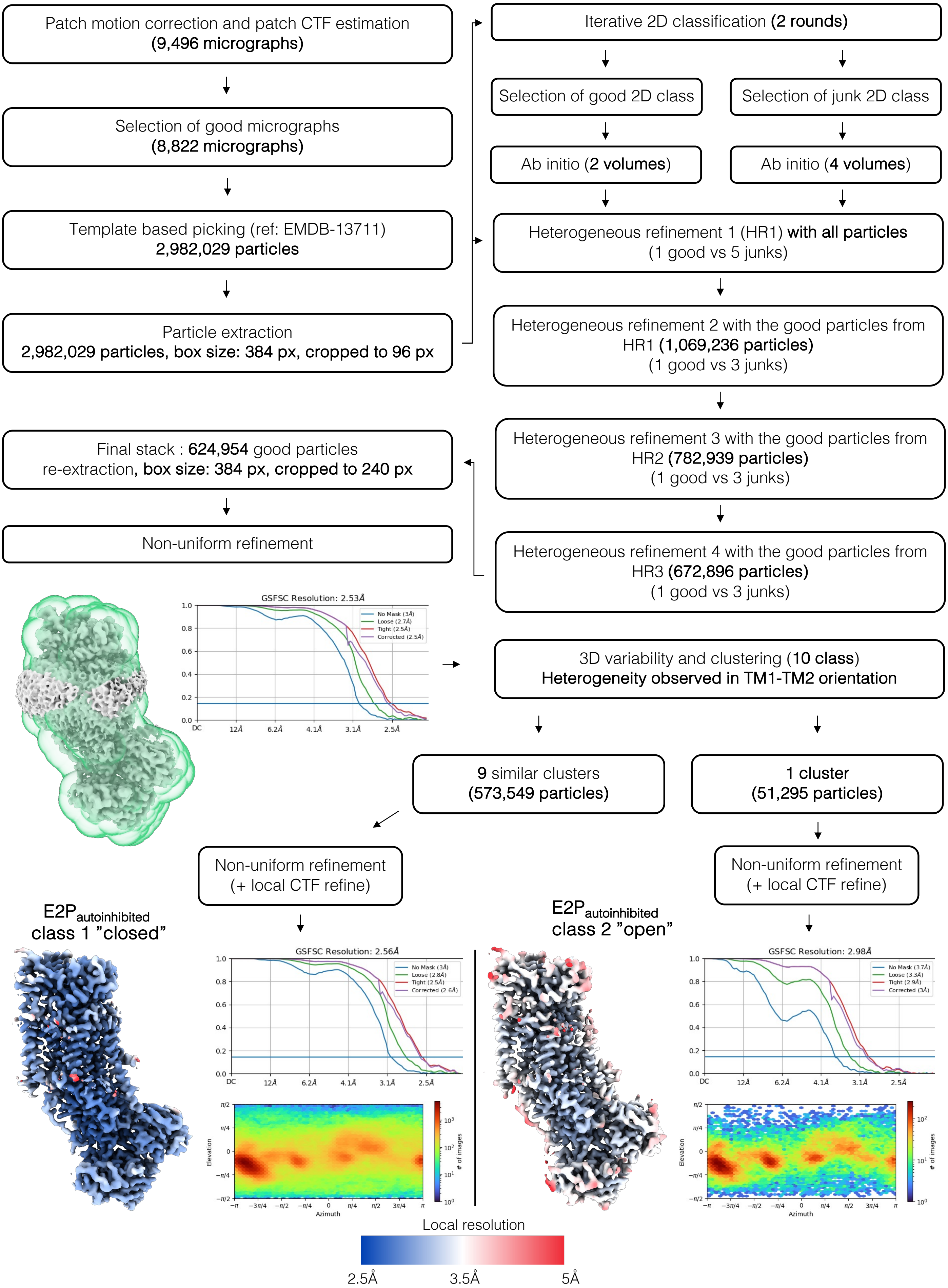

Supplementary Figure 7 – Cryo-EM data processing pipeline of ATP8B1-CDC50A ($E2P_{autoinhibited}$ "open" and "closed" conformations).

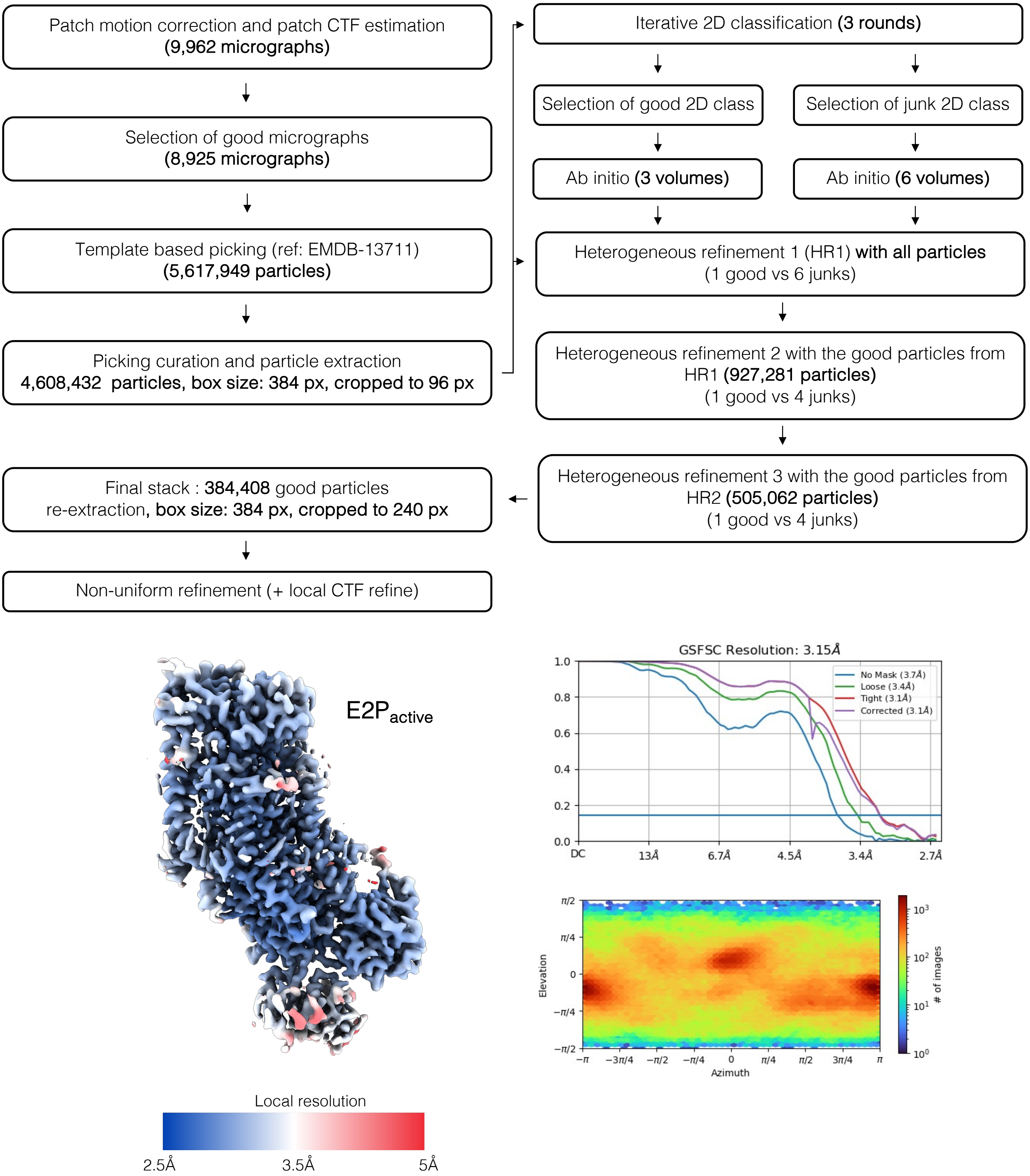

Supplementary Figure 8 – Cryo-EM data processing pipeline of ATP8B1-CDC50A ($E2P_{active}$).

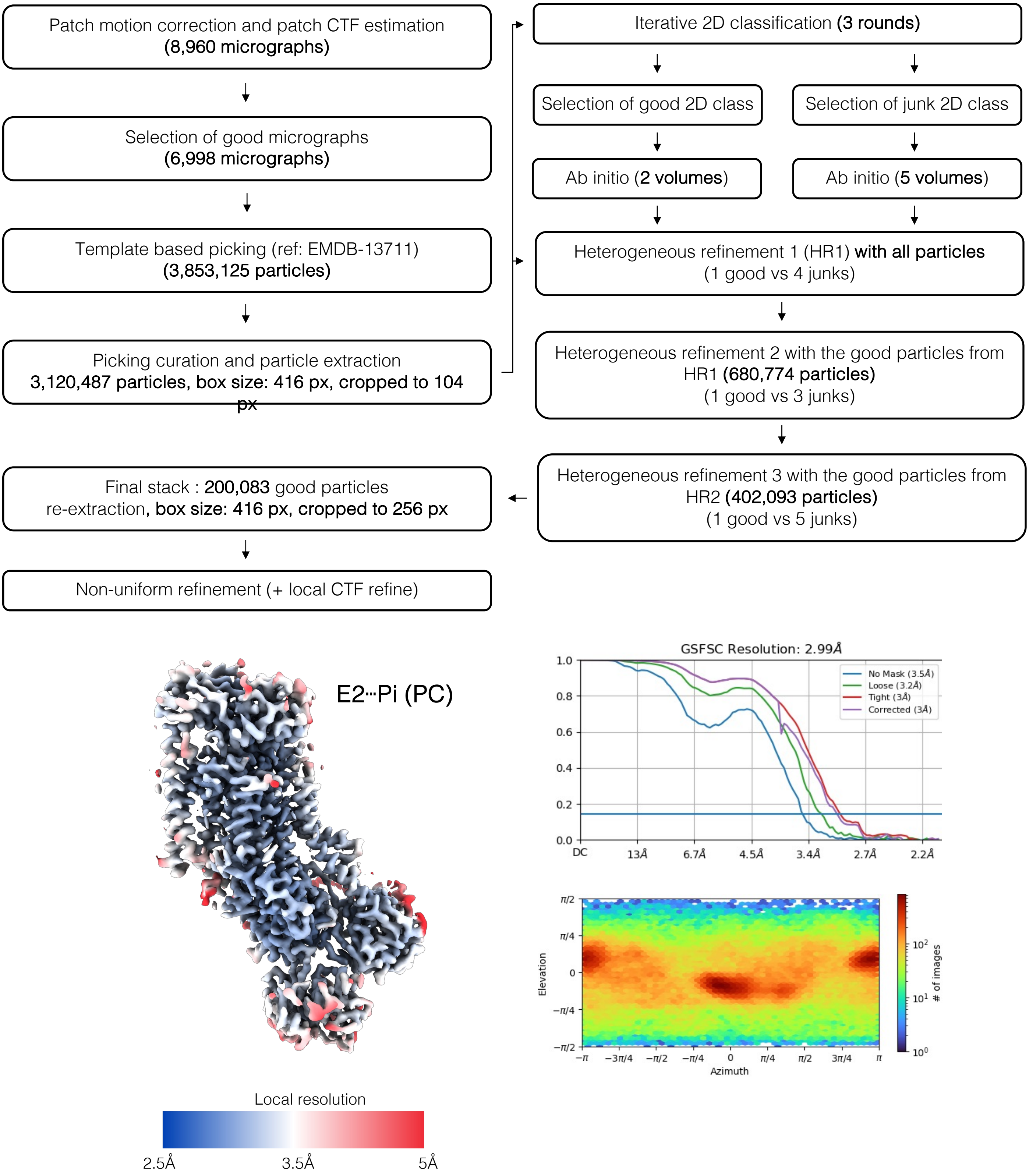

Supplementary Figure 9 – Cryo-EM data processing pipeline of ATP8B1-CDC50A (E2⋯Pi (PC)).

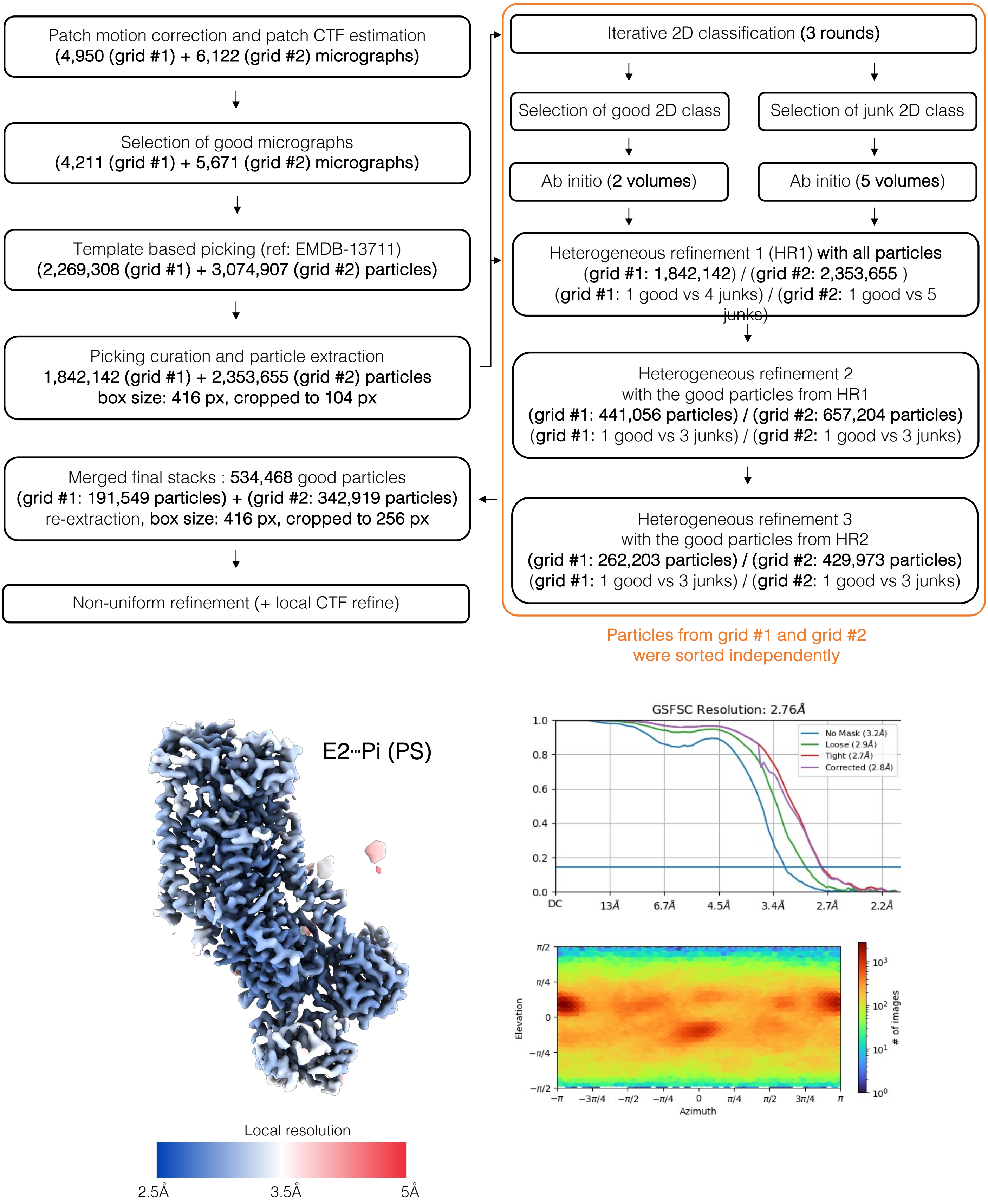

Supplementary Figure 10 – Cryo-EM data processing pipeline of ATP8B1-CDC50A (E2⋯Pi (PS))

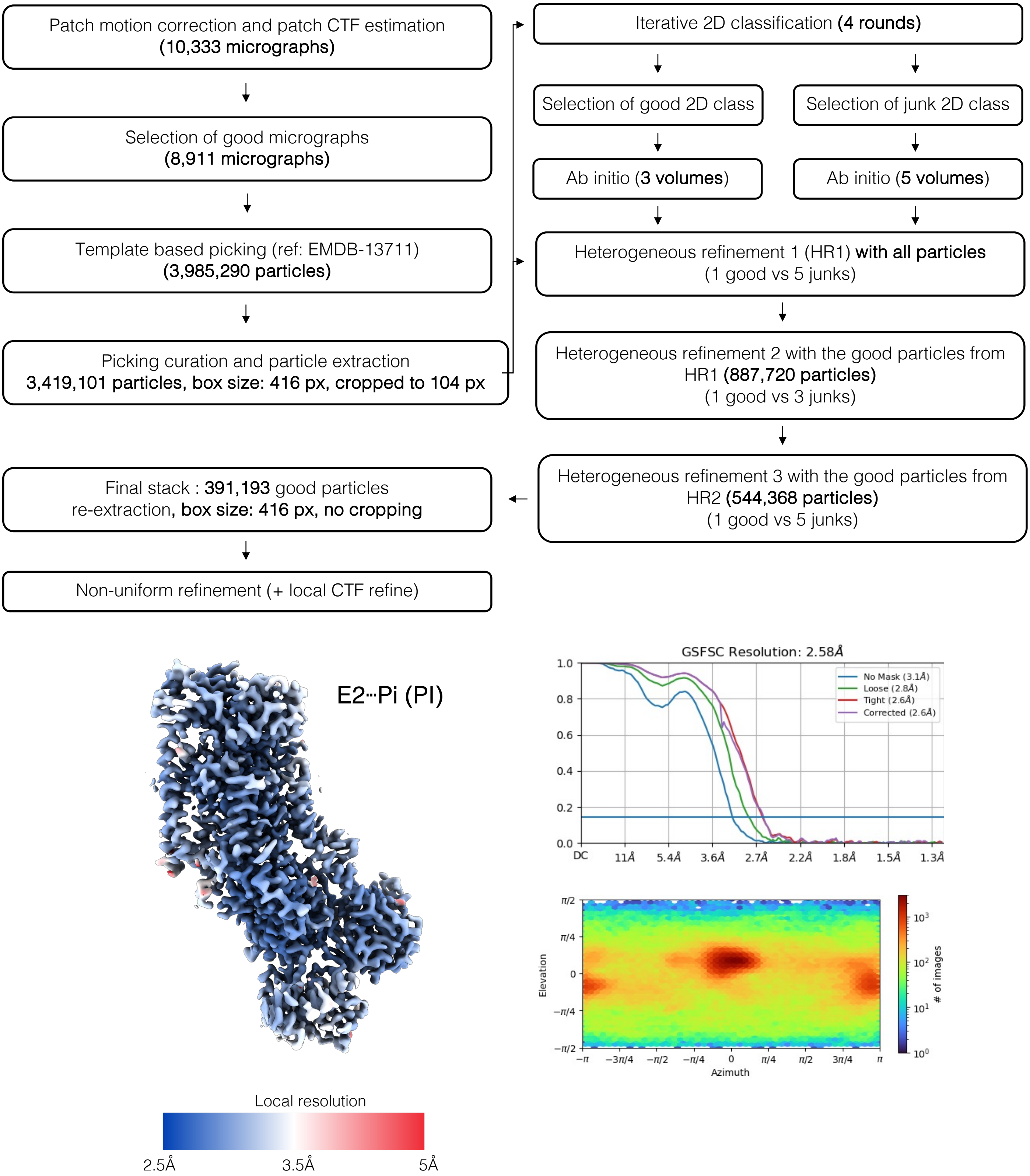

Supplementary Figure 11 – Cryo-EM data processing pipeline of ATP8B1-CDC50A (E2···Pi (PI)).

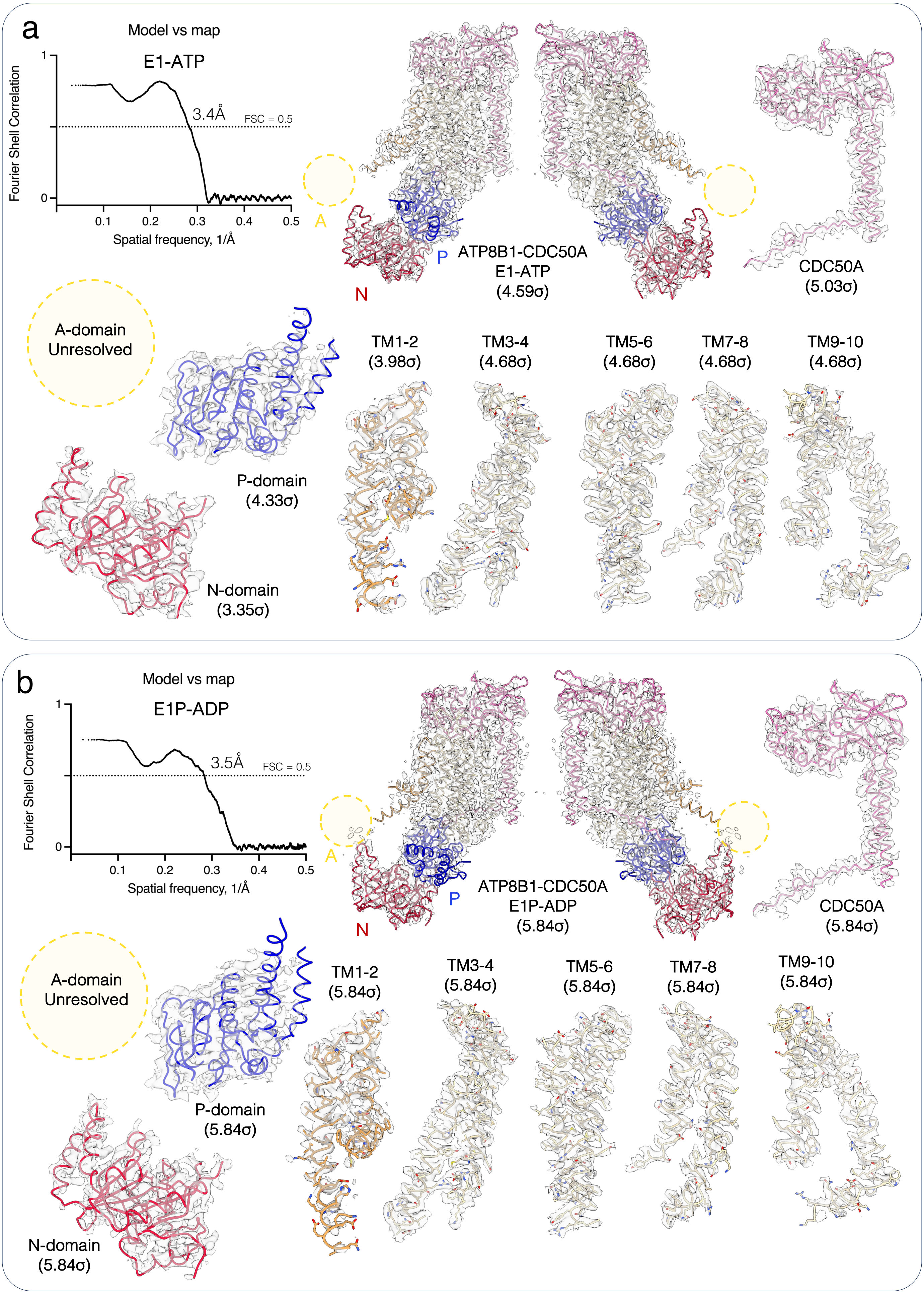

Supplementary Figure 12 – Map quality of (a) E1-ATP and (b) E1P-ADP states of ATP8B1-CDC50A

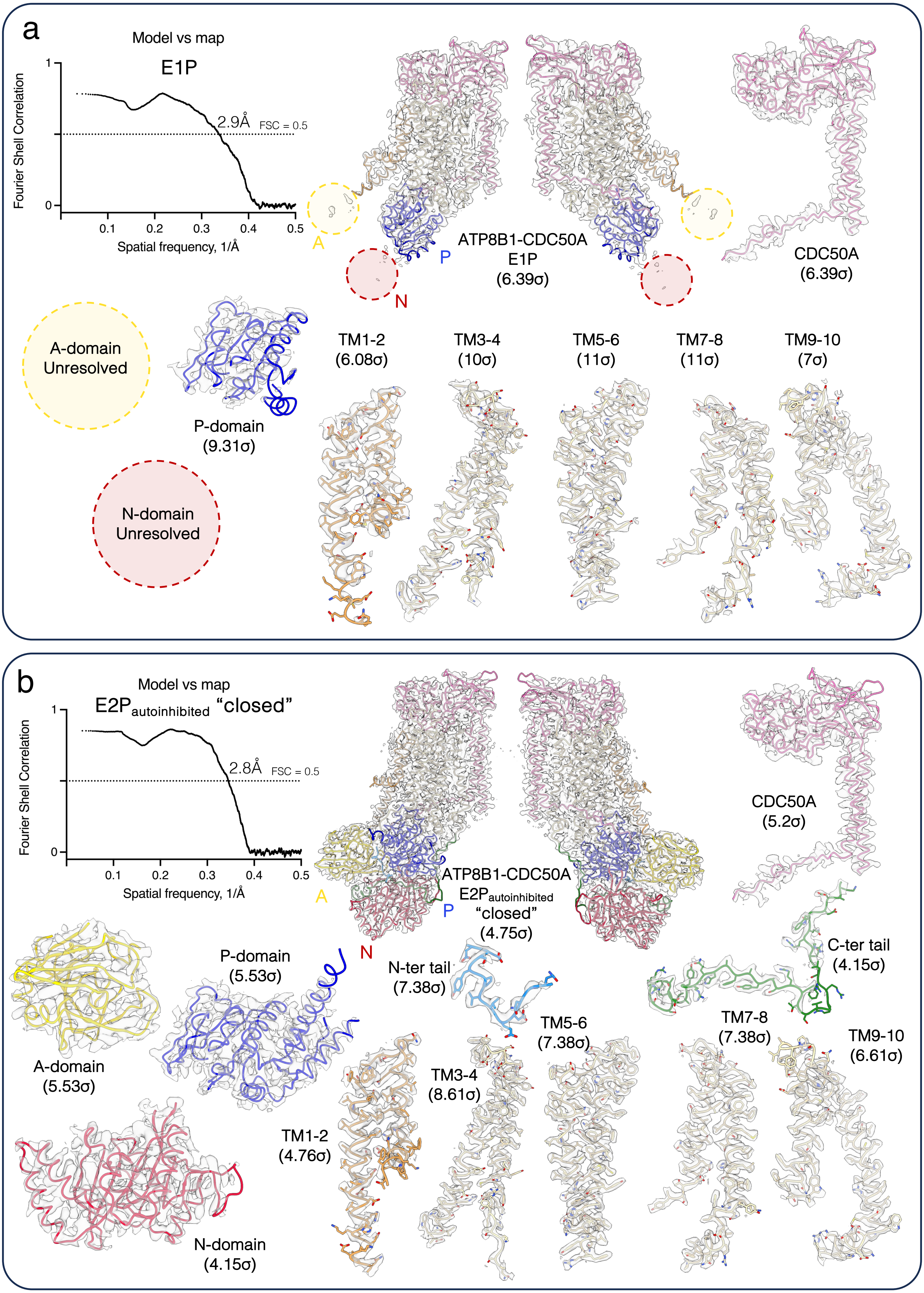

Supplementary Figure 13 – Map quality of (a) E1P and (b) $E2P_{autoinhibited}$ "closed" states of ATP8B1-CDC50A

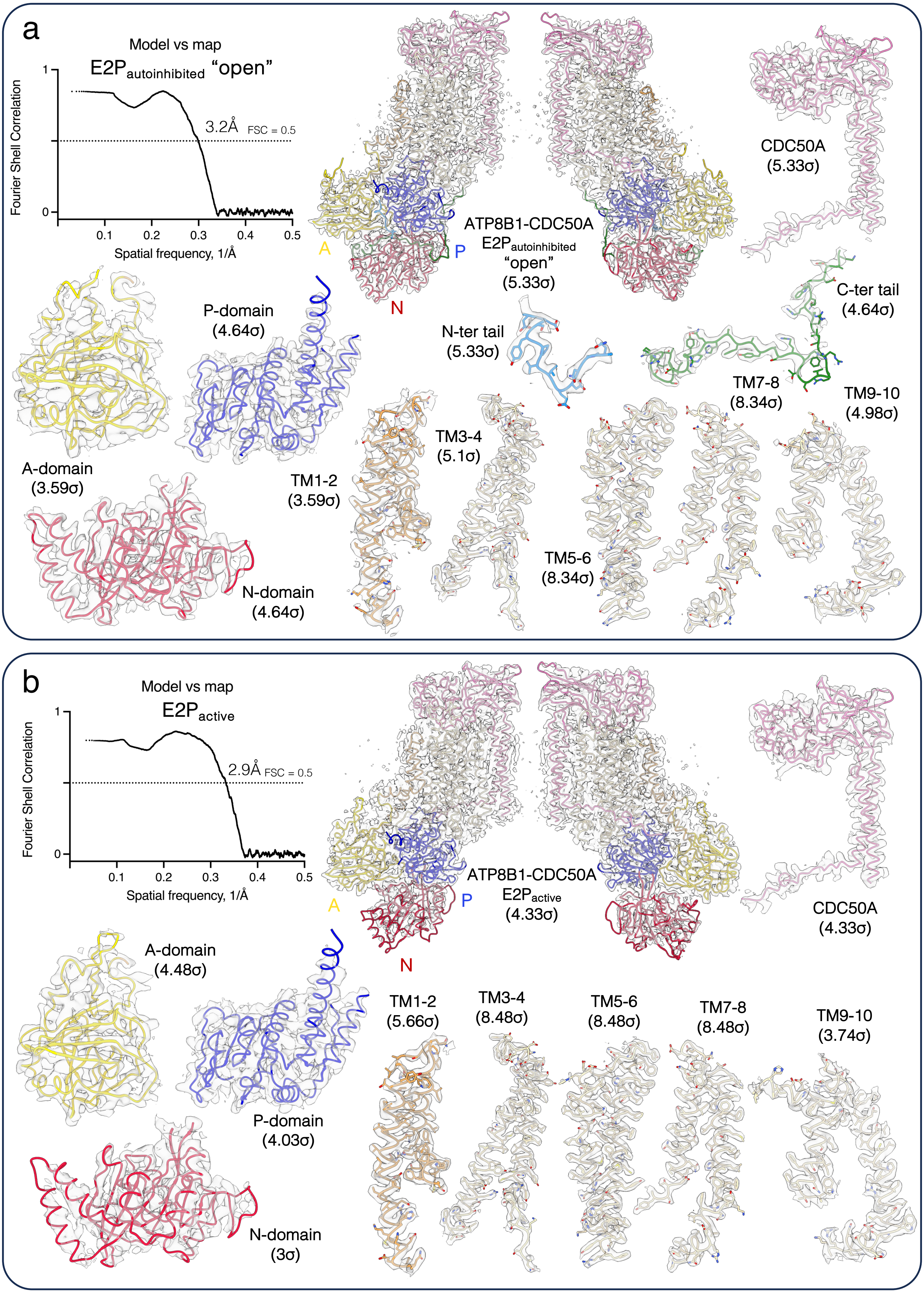

Supplementary Figure 14 – Map quality of (a) $E2P_{autoinhibited}$ "closed" and (b) $E2P_{active}$ states of ATP8B1-CDC50A.

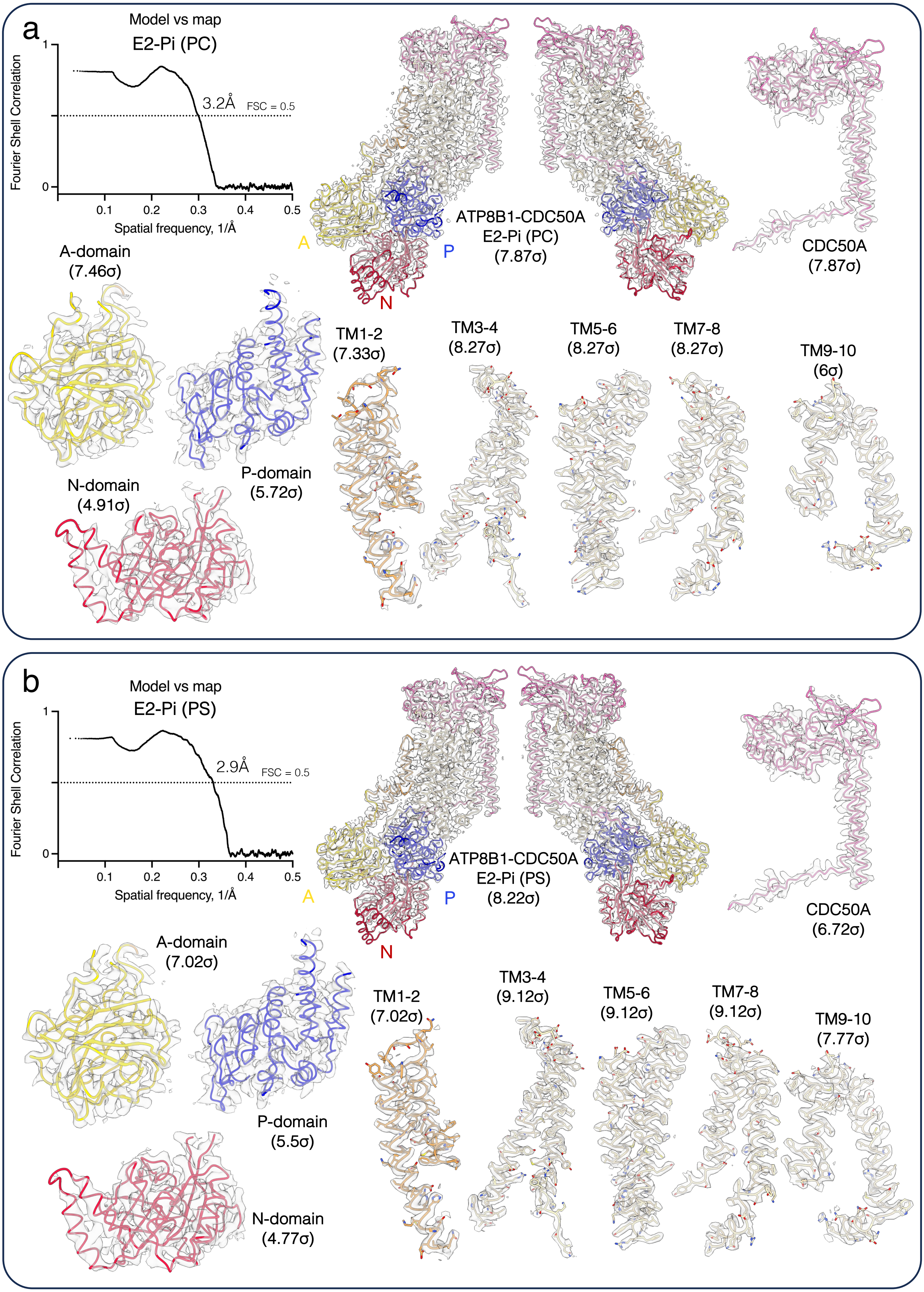

Supplementary Figure 15 – Map quality of (a) E2-Pi (PC) and (b) E2-Pi (PS) states of ATP8B1-CDC50A.

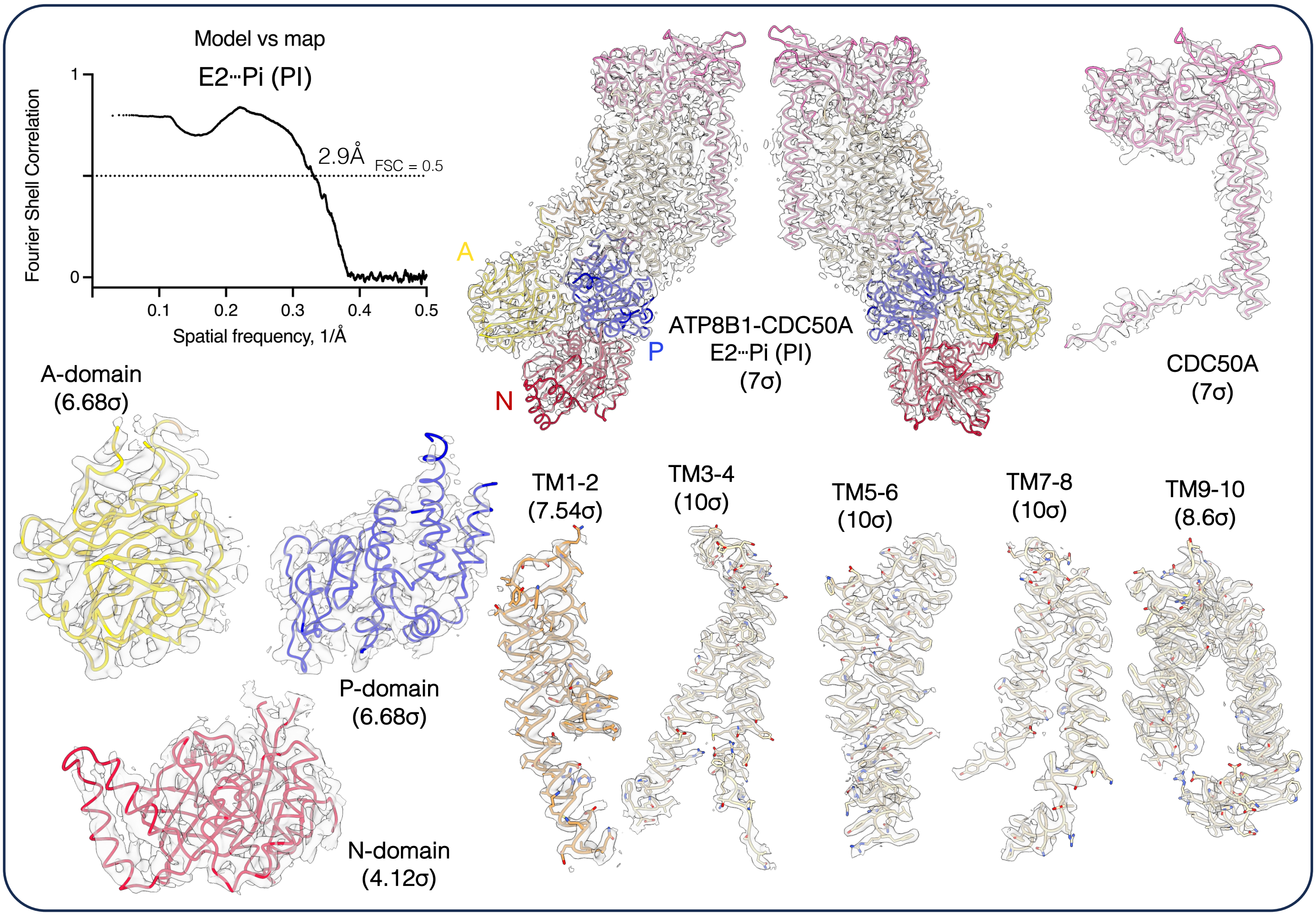

Supplementary Figure 16 – Map quality of the E2-Pi (PI) state of ATP8B1-CDC50A.

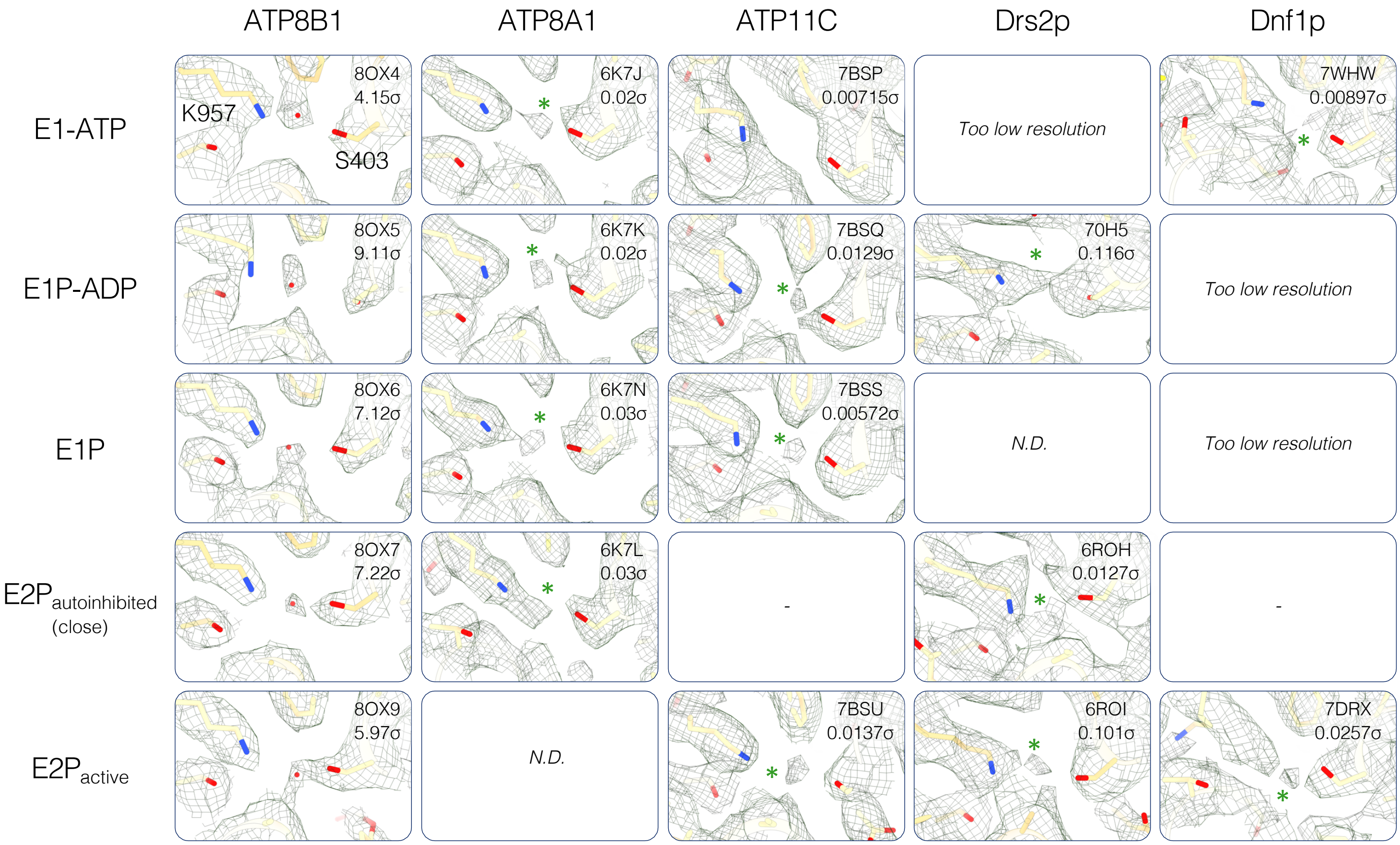

Supplementary Figure 17 – Water molecule EM density in the transport site of P4-ATPases.

Comparison of the cryo-EM maps of P4-ATPase in E1-ATP, E1P-ADP, E1P and E2P states. The water molecule density is highlighted by a green asterisk. For some P4-ATPases, the resolution was too low to compare the geometry of the side chains ("too low resolution"), and for some the structures have not been determined (N.D.) or do not exist (-). PDB codes: ATP8B1(this study: 8OX4, 8OX5, 8OX6, 8OX7, 8OX9); ATP8A1 (6K7J, 6K7K, 6K7N, 6K7L); ATP11C (7BSP, 7BSQ, 7BSS, 7BSU); Drs2p (7OH5, 6ROH, 6ROI); Dnf1p (7WHW, 7DRX).

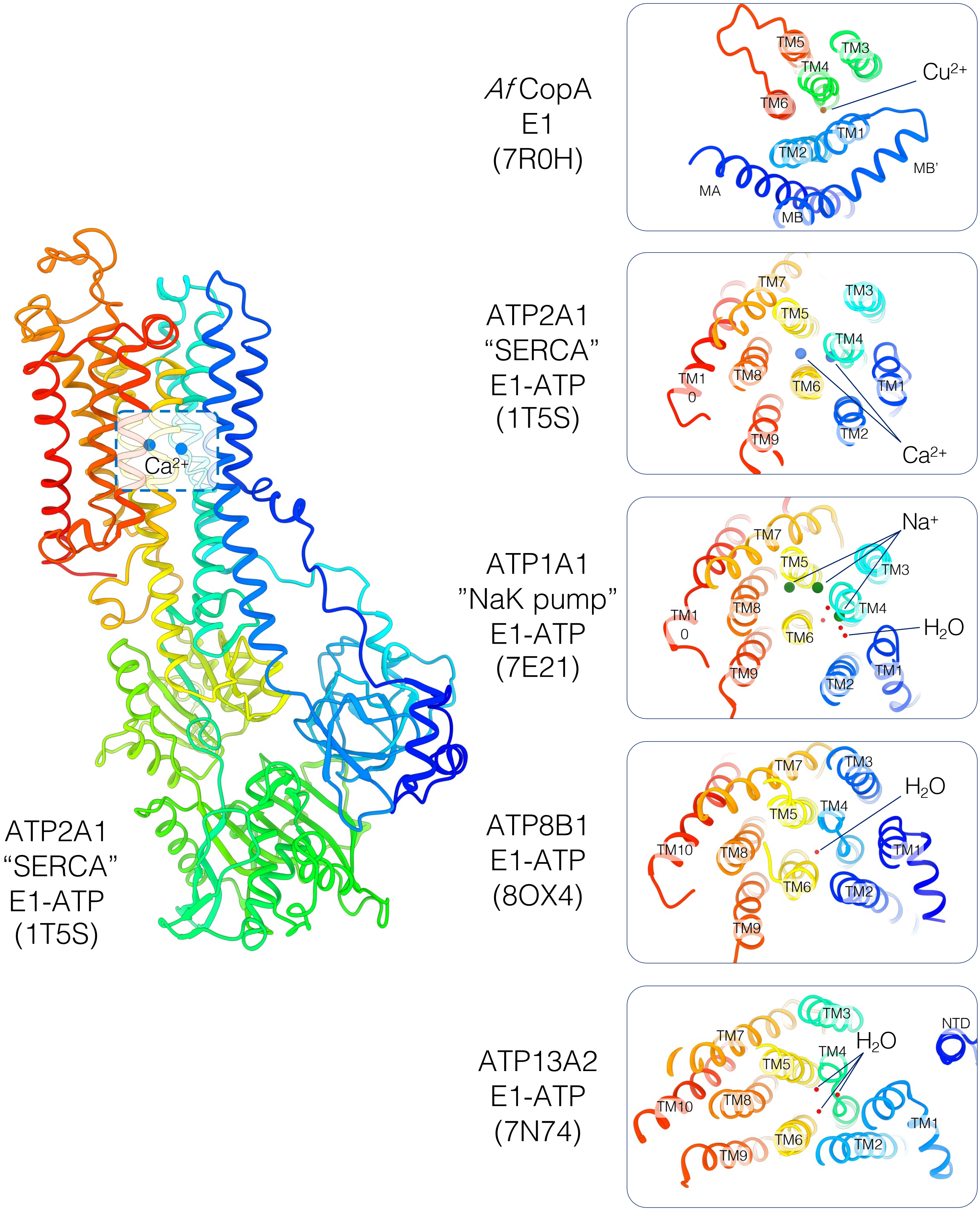

Supplementary Figure 18 – Comparison of the canonical transport site in different P-type ATPases in E1 or E1-ATP states. The comparison of the position of the water molecules observed in P4- and P5-ATPAse (ATP8B1 and ATP13A2) is similar to the position observed for cations in P1- and P2-ATPases (CopA, ATP2A1 and ATP1A1).

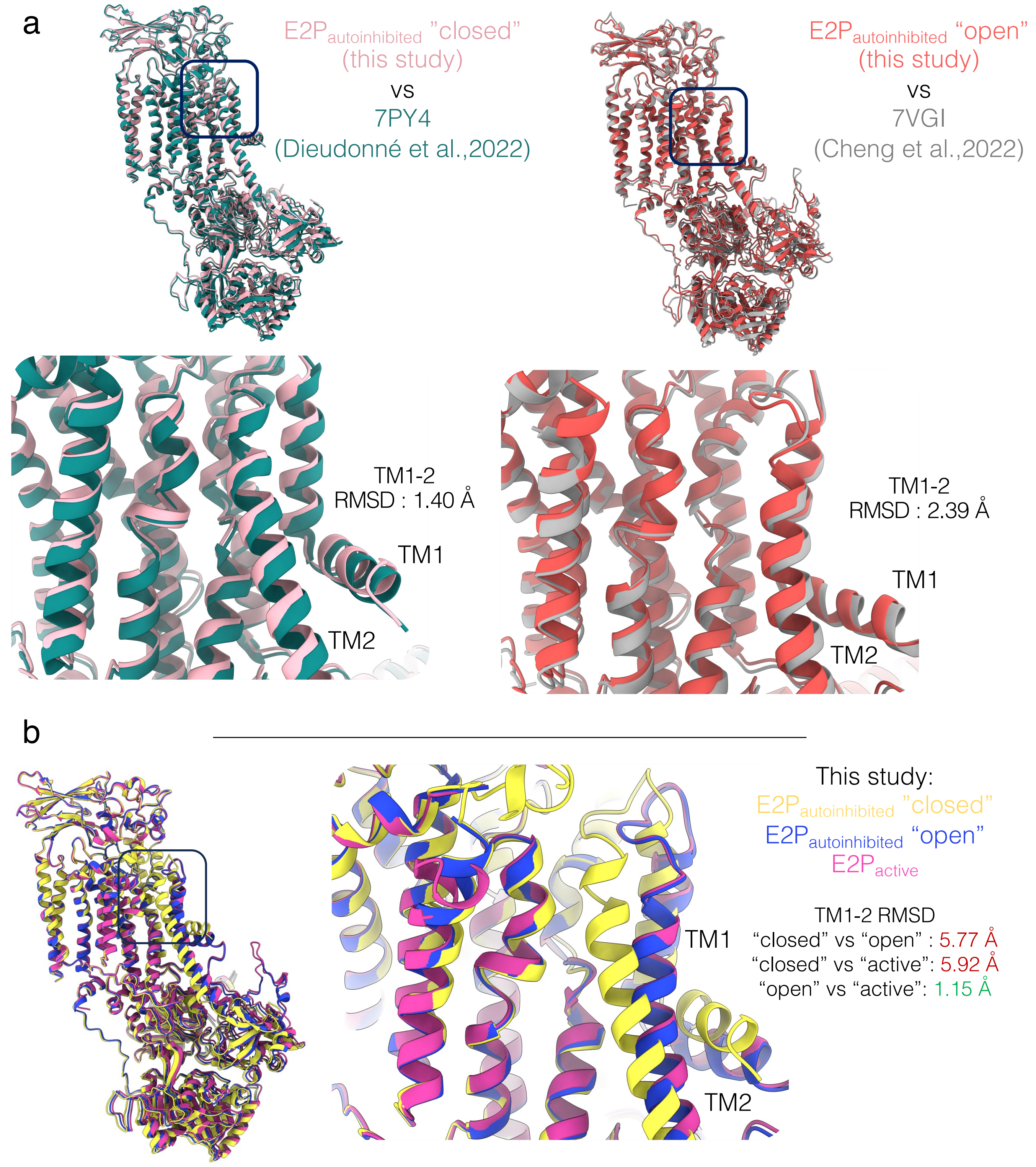

Supplementary Figure 19 – Comparison with the previously published structures of ATP8B1-CDC50A autoinhibited states.

a) ATP8B1-CDC50A in the $E2P_{autoinhibited}$ "closed" conformation (left) is similar to the previously published structure by Dieudonné et al., 2022. ATP8B1-CDC50A in the $E2P_{autoinhibited}$ "open" conformation (right) is similar to the previously published structure by Cheng et al., 2022. **b)** Structural alignment of the three E2P conformations described in this study. $E2P_{autoinhibited}$ "open" and the E2P active show a similar TM1-2 open conformation.

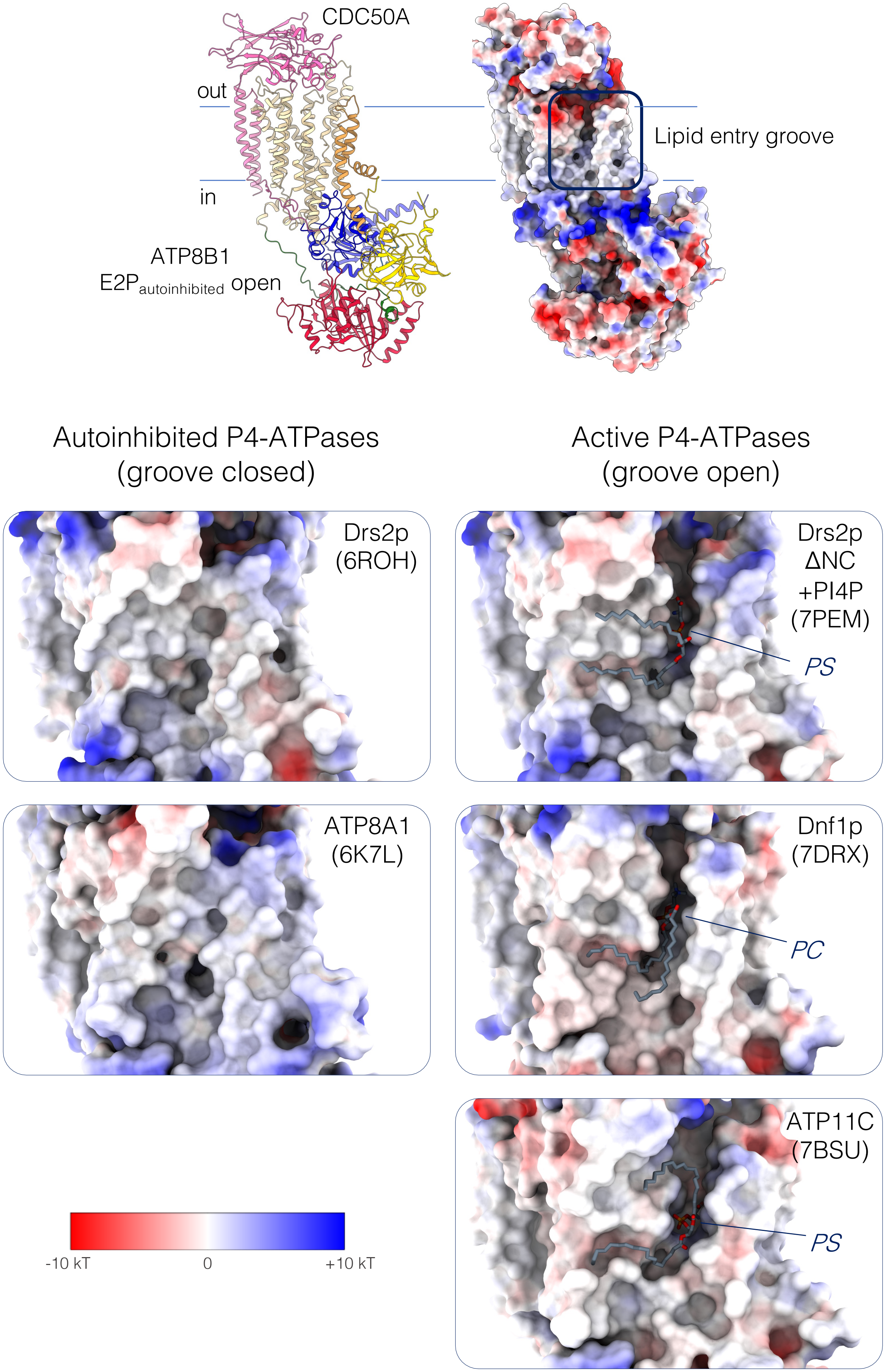

Supplementary Figure 20 – Comparison of the lipid groove of autoinhibited and active P4-ATPases in E2P state. The structure of the autoinhibited P4-ATPases in the E2P state display a close lipid entry groove while active proteins are open and filled with lipid substrate.

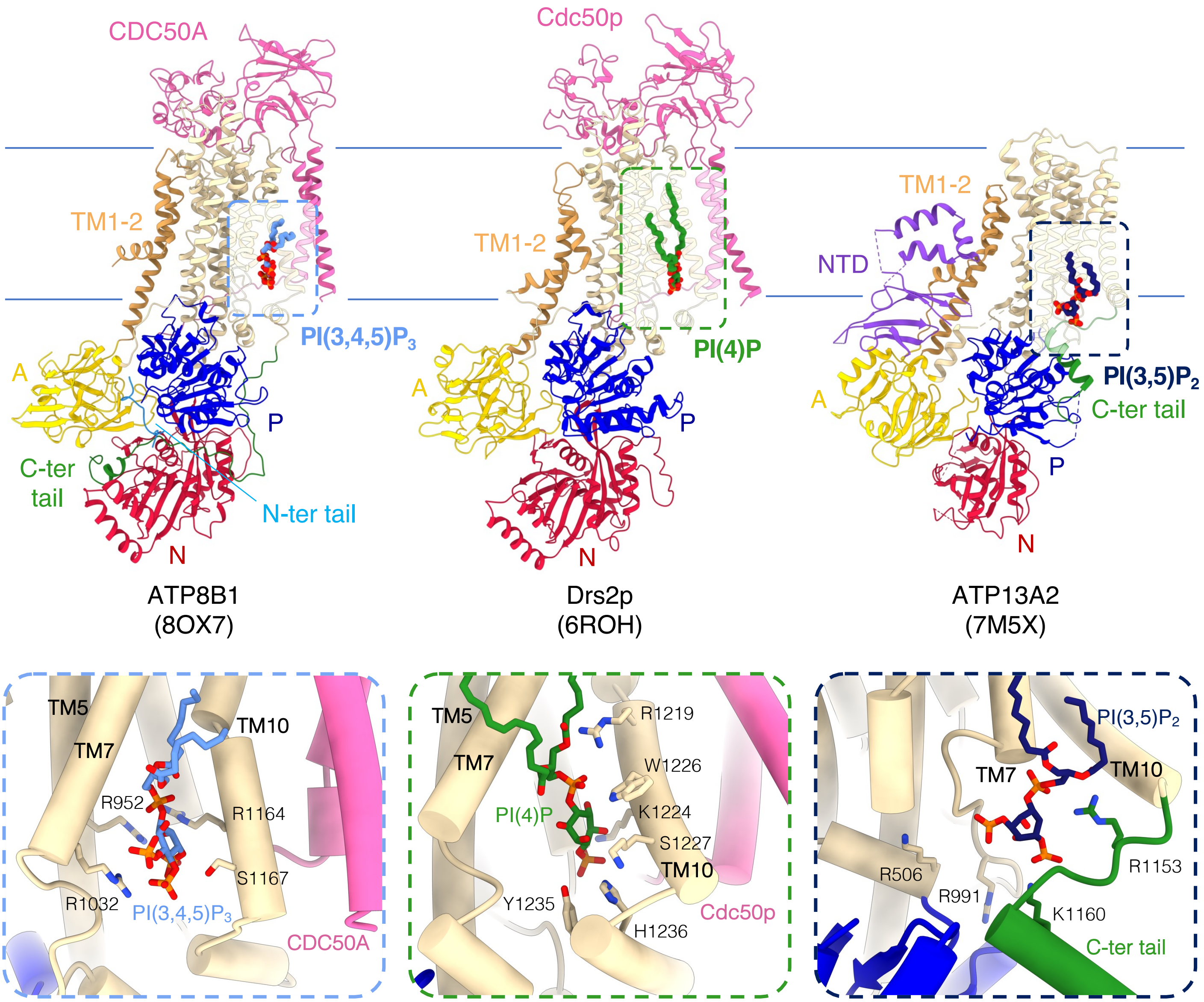

Supplementary Figure 21 – Structural comparison of the different phosphoinositide binding site observed in two P4-ATPases, ATP8B1 and Drs2p and in the P5-ATPase ATP13A2. The comparison of the phosphoinositide binding site observed in other P-type ATPases (Drs2 and ATP13A2) reveals that all phosphoinositides bind a similar region of the protein located in a cavity formed by TM7 and TM10 (and TM5 for P4-ATPases) filled with positively charged residues.

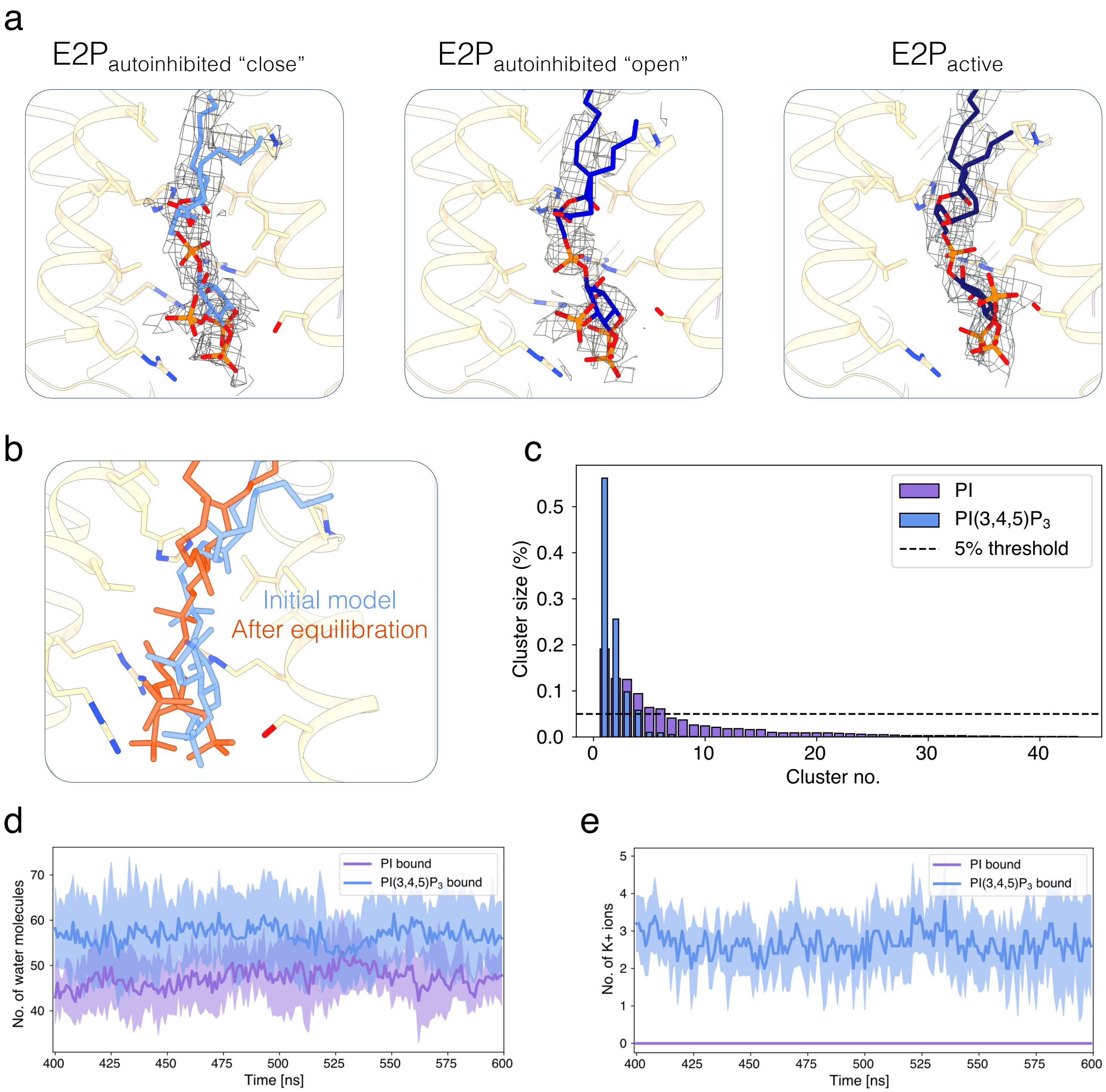

Supplementary Figure 22 – PI(3,4,5)P_3 binding site in other E2P conformations and complementary analysis of the MD data.

a) PI(3,4,5)P_3 binding site and the lipid associated EM density in the three E2P states of ATP8B1. **b)** Comparison of PI(3,4,5)P_3 position before (blue) and after (orange) the initial MD equilibration step. **c)** All Clusters of inositol ring coordinates from the last 200 ns of each trajectory with Daura's algorithm for PI(3,4,5)P_3 (blue) and PI (purple). **d)** Hydration of the lipid binding site. The average number of water molecules present in the lipid binding site for the PI(3,4,5)P_3-bound system (blue) and the PI-bound (purple) over the last 200 ns of the simulations. The shaded area indicates the standard deviation over the five replicates of each system. **e)** K^+ ions surrounding the lipid headgroup. The average number of K^+ ions present within 6 Å of the PI(3,4,5)P_3 and the PI (purple) headgroup (blue) over the last 200 ns of the simulations. The shaded area indicates the standard deviation over the five replicates of each system.

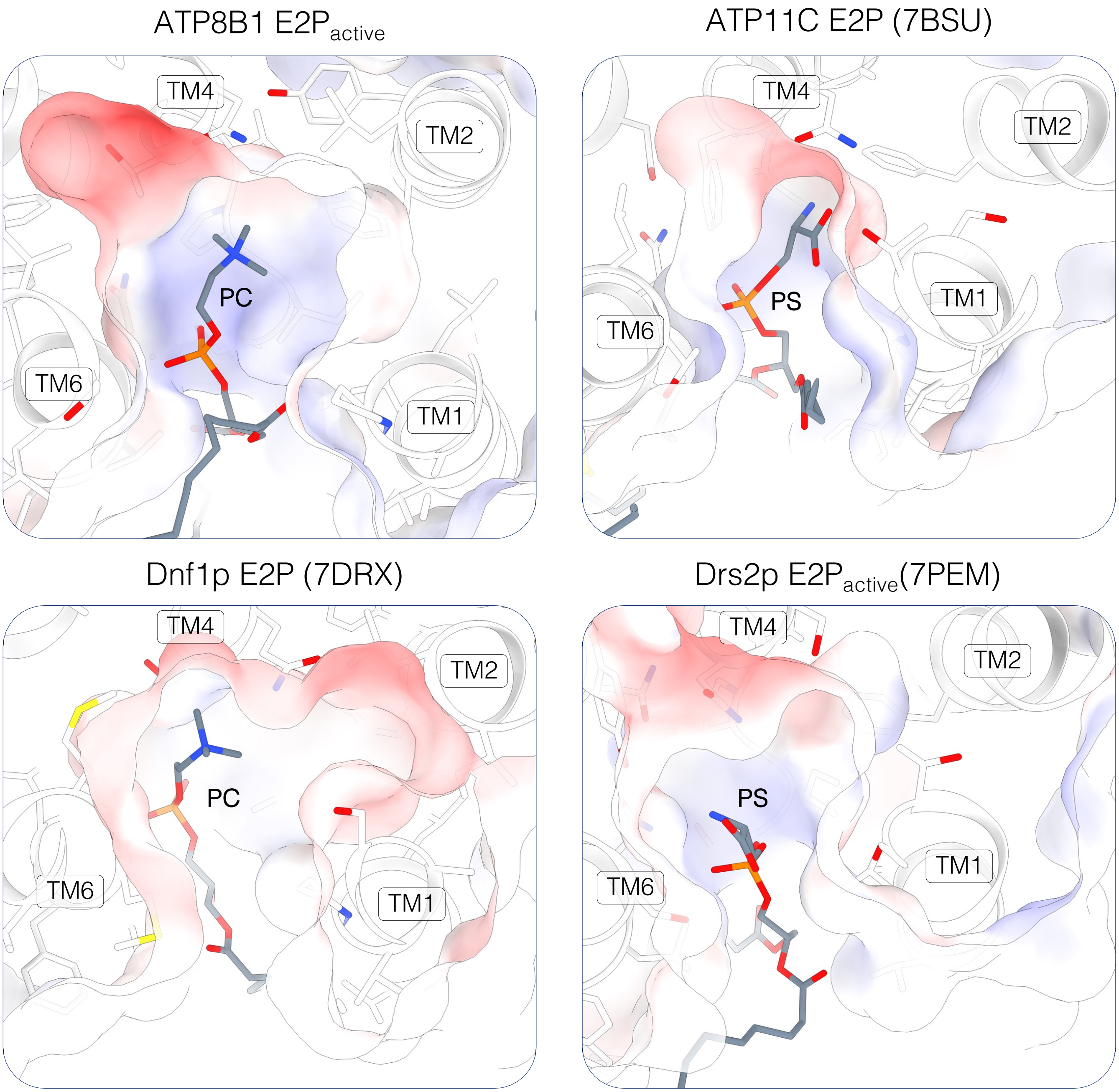

Supplementary Figure 23 – Comparison of the lipid binding site in P4-ATPases in E2P$_{active}$ conformation.

Exoplasmic view of the transport lipid binding site of different P4-ATPase in E2P$_{active}$ conformation. The surface around the transport lipid is shown as electrostatic surface.

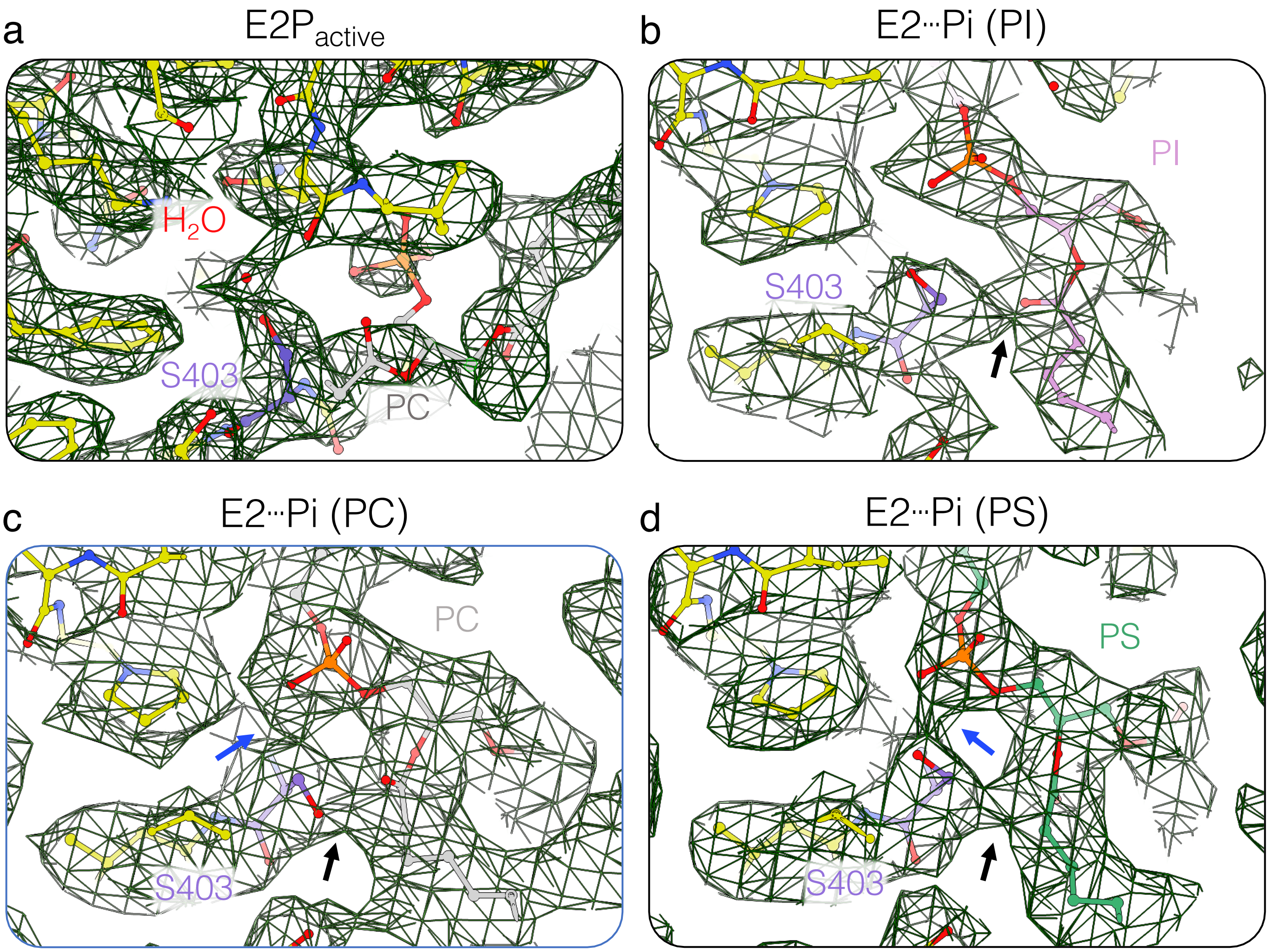

Supplementary Figure 24 – Close up view of the transport lipid binding site in the E2P $_{active}$ and E2···Pi (lipid occluded) conformation and their associated electron map.

a) E2P active conformation (contour map: 5.7σ), **b)** E2···Pi (PI) conformation (contour map: 4.34σ step 2), **c)** E2···Pi (PC) conformation (contour map: 3.3σ), **d)** E2···Pi (PS) conformation (contour map: 4.3σ). In **b, c** and **d**, arrows indicated continuous densities observed between the possible different rotamers of the S403 and the phosphate of (blue arrow) and the sn2-ester bond of the lipid (black arrow).

a

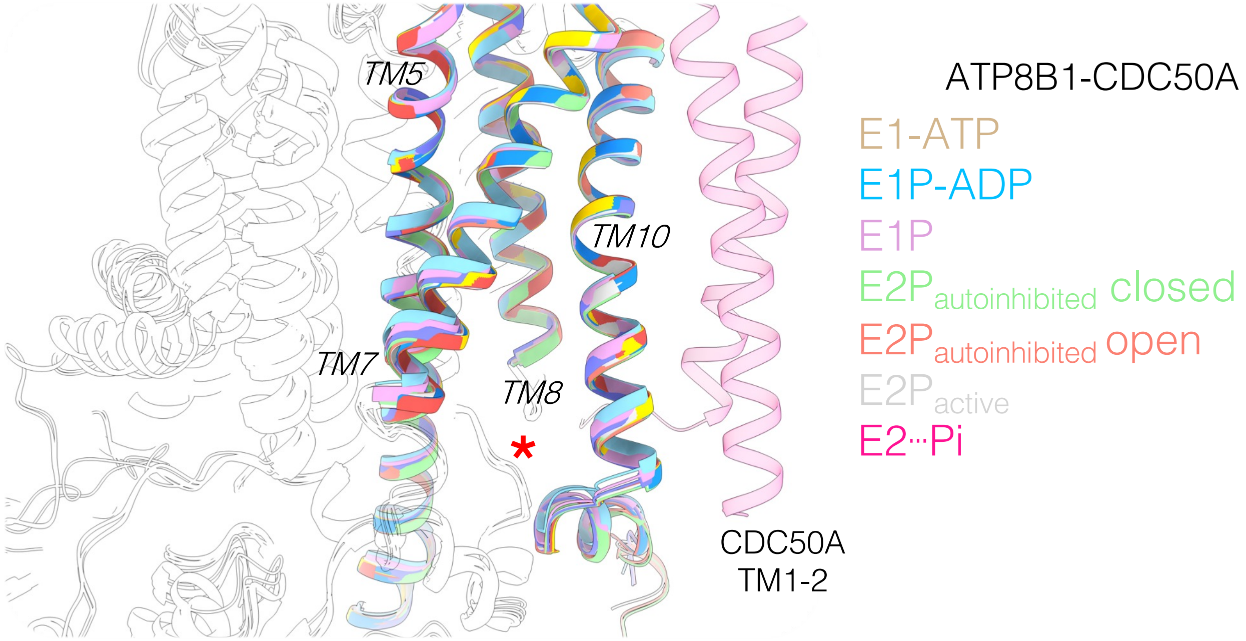

b

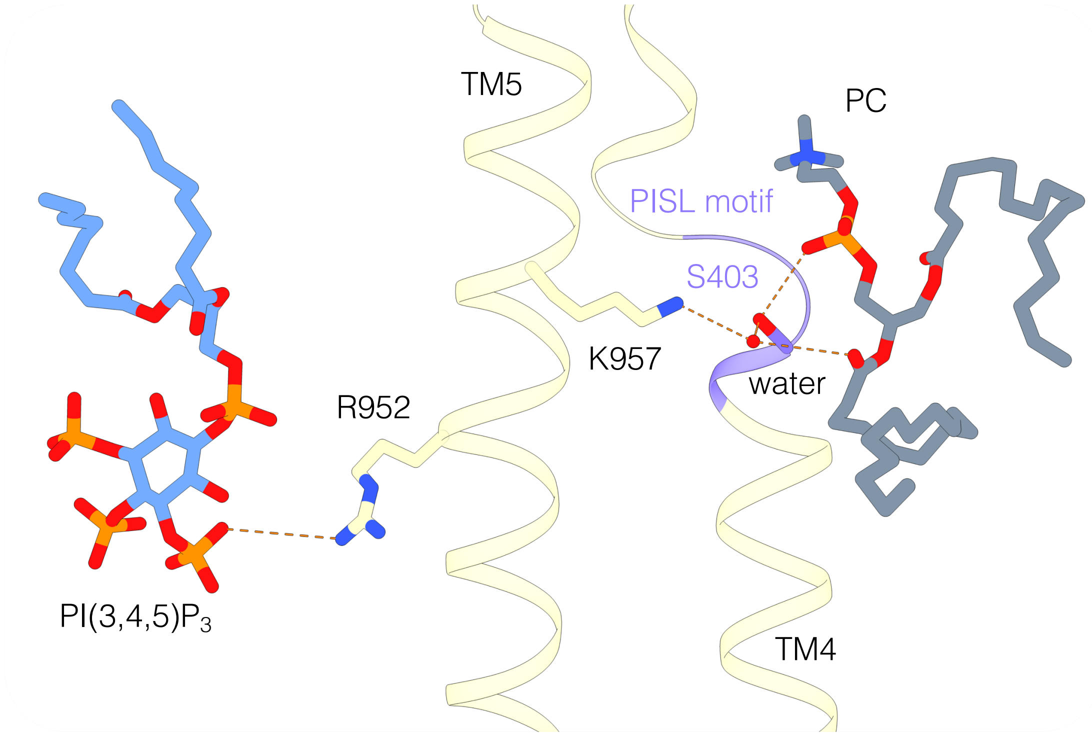

Supplementary Figure 25 – PI(3,4,5)P_3 binding site in the different ATP8B1-CDC50A conformations reported in this study and its putative link with the lipid transport site.
a) Comparison of the PI(3,4,5)P_3 binding site (red asterisk) conformation in the different structures presented in this study**. b)** Close-up view of the PI(3,4,5)P_3 binding site and the transport lipid binding (PC) site in the $E2P_{active}$ conformation. PI(3,4,5)P_3 tightly interacts with R952 on TM5 adjacent to the lipid transport site. For clarity purposes, only TM4 and TM5 are shown.

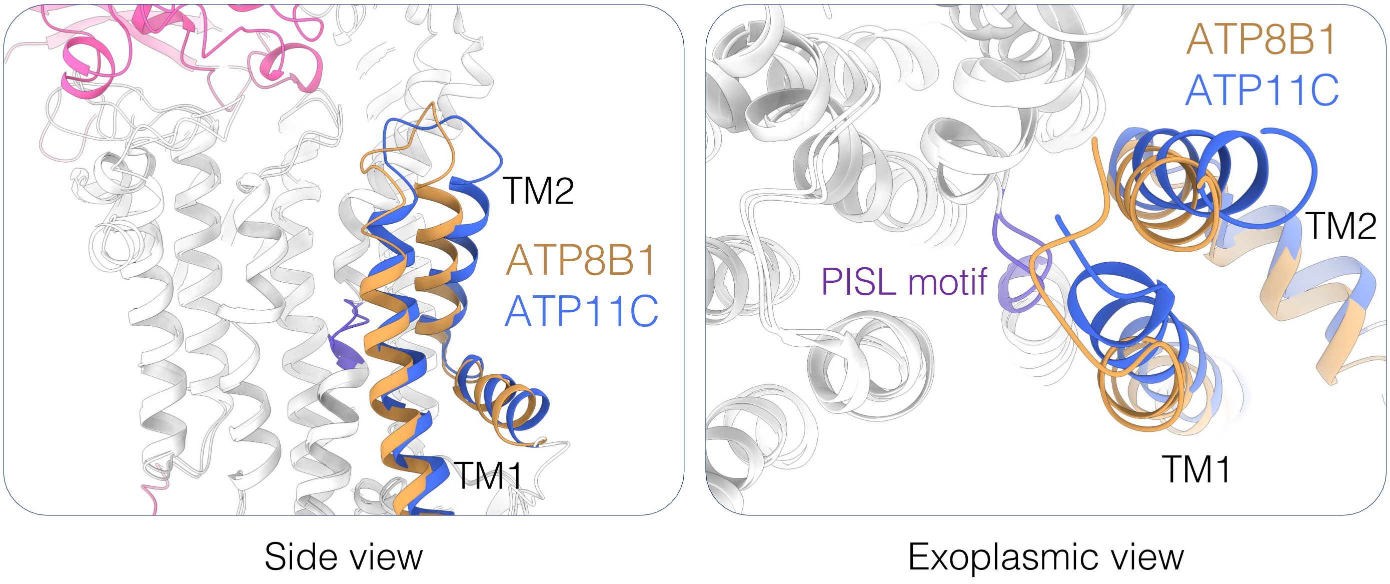

Supplementary Figure 26 – TM1-2 orientation in ATP8B1 and ATP11C in E2P$_{active}$ conformation.

ATP8B1 TM1-2 are shown in brown, ATP11C (PDB: 7BSU) TM1-2 are shown in blue. The lipid recognition motif (PISL) of TM4 is shown in purple. CDC50A (from the ATP8B1-CDC50A complex) is shown in pink.

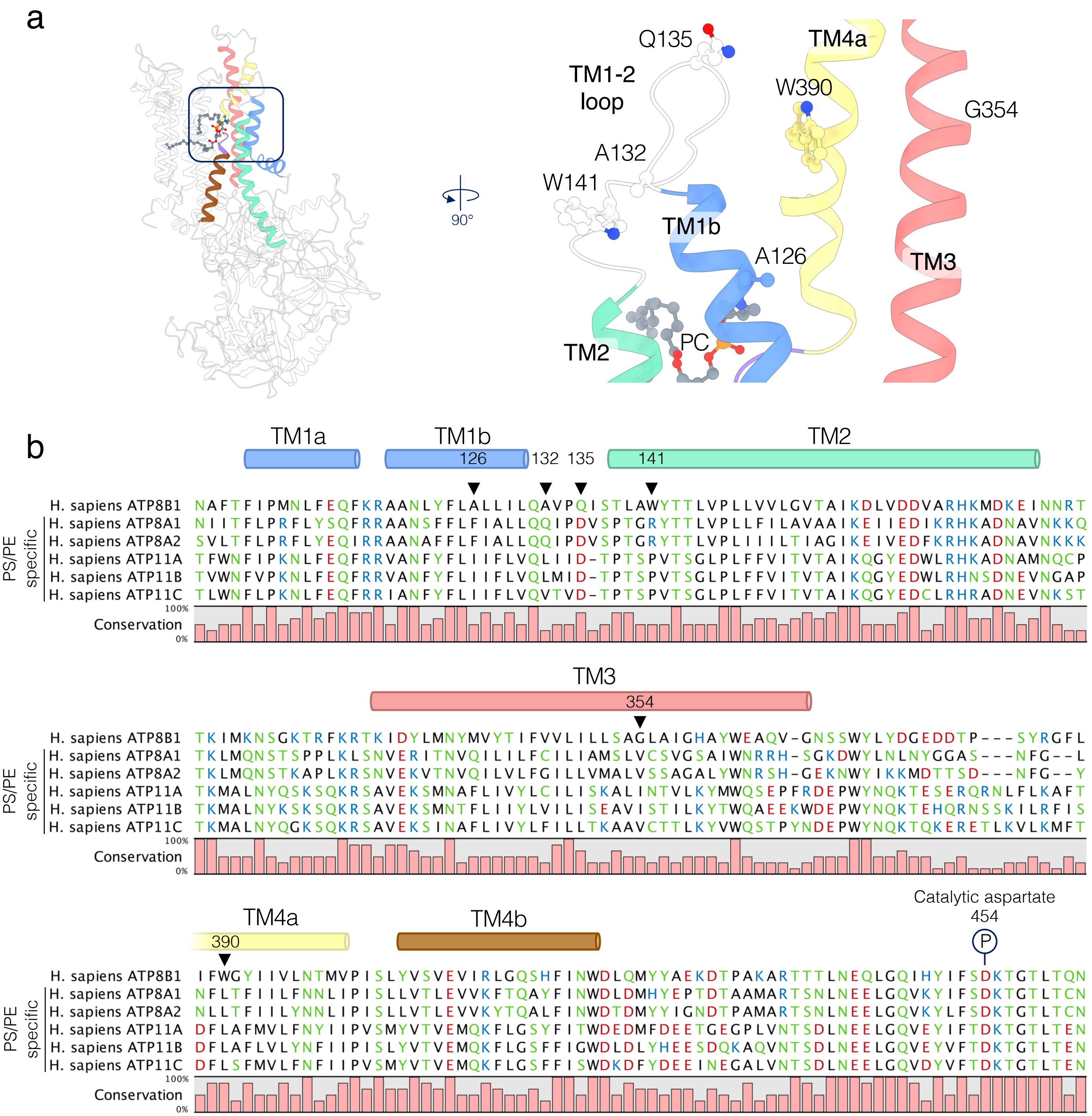

Supplementary Figure 27 – Sequence alignment of TM1-4 of ATP8B1 and PS-specific human P4-ATPases. a) ATP8B1 in the E2P$_{active}$ PC bound conformation with a close-up view of TM1-4 organization close to the lipid binding site. TM1, TM2, TM3 and TM4 are blue, turquoise, yellow and red, respectively. The non-conserved residues between ATP8B1 and PS-specific human P4-ATPases are shown as sticks. **b)** Sequence alignment of TM1-4 of ATP8B1 and PS-specific human P4-ATPases, colors as in a). (Uniprot ID: hATP8B1(O43520); hATP8A1 (Q9Y2Q0); hATP8A2 (Q9NTI2); hATP11A (P98196); hATP11B (Q9Y2G3); hATP11C (Q8NB49)).

	**E1-ATP** (EMDB-17256) (PDB 8OX4)	**E1P-ADP** (EMDB-17257) (PDB 8OX5)	**E1P** (EMDB-17258) (PDB 8OX6)
**Sample preparation**	ΔC-ter	ΔC-ter	ΔC-ter
Construct	ATP8B1-CDC50A	ATP8B1-CDC50A	ATP8B1-CDC50A
Ligands / Inhibitors	AMPPCP, PI(4,5)P_2	ADP, AlF_x, PI(4,5)P_2	AlF_x, PI(4,5)P_2
**Data collection and processing**			
Magnification		130,000x	
Voltage (kV)		300	
Microscope		Titan Krios G3i	
Camera		Gatan K3	
Physical pixel size (Å/pix)		0.647	
Electron exposure (e–/Å^2)		60	
Defocus range (µm)		0.7-1.9	
Number of movies	7,212	11,168	10,640
Initial particle images (no.)	2,486,972	3,571,944	4,207,324
Final particle images (no.)	179,570	226,859	813,354
Symmetry imposed	C1	C1	C1
Map resolution (Å)	3.15	2.9	2.39
FSC threshold	0.143	0.143	0.143
**Refinement**			
Initial model used (PDB code)	7PY4	7PY4	7PY4
Model resolution (Å)	3.4	3.5	2.9
FSC threshold	0.5	0.5	0.5
Map sharpening *B* factor (Å^2)	-100	-75	-83
Model composition			
Non-hydrogen atoms	9756	9755	7358
Protein residues	1198	1197	903
Water	1	1	1
Ligands	1 ACP, 1 MG, 4 NAG, 1 BMA	1 AlF, 1 ADP 1 MG, 4 NAG, 1 BMA	1 AlF, 1 ADP 1 MG, 4 NAG, 1 BMA
B factors (Å^2, min/max/mean)	5.77/93.41/37.46	10.77/90.03/42.76	37.28/116.43/961.7
Protein	18.68/61.96/43.95	33.22/80.69/49.42	5
Ligand	13.95/13.95/13.95	14.64/14.64/14.64	56.61/90.46/70.17
Water			42.01/42.01/42.01
R.m.s. deviations			
Bond lengths (Å)	0.003	0.003	0.003
Bond angles (°)	0.509	0.537	0.580
Validation			
MolProbity score	1.51	1.91	1.79
Clashscore	5.65	9.72	7.88
Ramachandran plot			
Favored (%)	96.79	94.01	94.74
Allowed (%)	3.21	5.99	5.26
Outliers (%)	0.0	0.0	0.0

Supplementary Table 1 - Data collection and refinement statistics

	$E2P_{autoinhibited}$ "closed" (EMDB-17259) (PDB 8OX7)	$E2P_{autoinhibited}$ "open" (EMDB-17260) (PDB 8OX8)	$E2P_{active}$ (EMDB-17261) (PDB 8OX9)
**Sample preparation**			
Construct	Full-length ATP8B1-CDC50A	Full-length ATP8B1-CDC50A	ΔC-ter ATP8B1-CDC50A
Ligands / Inhibitors	ATP, PI(3,4,5)P_3	ATP, PI(3,4,5)P_3	BeF_x, PI(3,4,5)P_3, POPC
**Data collection and processing**			
Magnification	130,000x		
Voltage (kV)	300		
Microscope	Titan Krios G3i		
Camera	Gatan K3		
Physical pixel size (Å/pix)	0.647		
Electron exposure (e–/Å^2)	60		
Defocus range (μm)	0.7-1.9		
Number of movies	9,496		9,962
Initial particle images (no.)	2,982,029		5,471,793
Final particle images (no.)	573,549	51,295	256,001
Symmetry imposed	C1	C1	C1
Map resolution (Å)	2.56	2.98	2.72
FSC threshold	0.143	0.143	0.143
**Refinement**			
Initial model used (PDB code)	7PY4	7PY4	7PY4
Model resolution (Å)	2.8	3.2	2.9
FSC threshold	0.5	0.5	0.5
Map sharpening *B* factor (Å^2)	-87	-66	-93
Model composition			
Non-hydrogen atoms	11756	11874	11583
Protein residues	1440	1452	1411
Water	1	1	9
Ligands	1 IP9,1 MG, 4 NAG, 1 BMA	1 IP9,1 MG, 4 NAG, 1 BMA	1 IP9, 1 MG, 1 POV, 1 BEF, 4 NAG, 1 BMA
B factors (Å^2, min/max/mean)	8.57/88.66/35.12	40.84/121.13/70.78	28.22/140.18/66.54
Protein	24.76/71.63/38.97	44.13/107.14/77.92	35.48/106.00/62.09
Ligand	16.49/16.49/16.49	53.89/53.89/53.89	34.57/47.42/39.46
Water			
R.m.s. deviations			
Bond lengths (Å)	0.003	0.004	0.003
Bond angles (°)	0.512	0.499	0.536
Validation			
MolProbity score	1.56	1.65	1.32
Clashscore	5.63	6.55	4.67
Ramachandran plot			
Favored (%)	96.28	95.82	97.64
Allowed (%)	3.72	4.18	2.36
Outliers (%)	0.0	0.0	0.0

Supplementary Table 1 - Data collection and refinement statistics

	E2···Pi (PC) (EMDB-17263) (PDB 8OXB)	E2···Pi (PS) (EMDB-17262) (PDB 8OXA)	E2···Pi (PI) (EMDB-17264) (PDB 8OXC)
**Sample preparation**	ΔC-ter	ΔC-ter	ΔC-ter
Construct	ATP8B1-CDC50A	ATP8B1-CDC50A	ATP8B1-CDC50A
Ligands / Inhibitors	VO_4, $PI(4,5)P_2$, POPC	VO_4, $PI(4,5)P_2$, POPS	VO_4, $PI(4,5)P_2$, soy PI
**Data collection and processing**			
Magnification		130,000x	
Voltage (kV)		300	
Microscope		Titan Krios G3i	
Camera		Gatan K3	
Physical pixel size (Å/pix)		0.647	
Electron exposure (e–/Å^2)		60	
Defocus range (µm)	0.7-1.9	0.7-1.9	0.7-1.9
Number of movies	8,960	4,950 (grid #1) 6,122 (grid #2)	10,333
Initial particle images (no.)	3,120,487	4,680,808	3,419,101
Final particle images (no.)	200,083	534,468	391.193
Symmetry imposed	C1	C1	C1
Map resolution (Å)	2.99	2.76	2.58
FSC threshold	0.143	0.143	0.143
**Refinement**			
Initial model used (PDB code)	7PY4	7PY4	7PY4
Model resolution (Å)	3.2	2.9	2.9
FSC threshold	0.5	0.5	0.5
Map sharpening *B* factor (Å^2)	-85	-98	-77
Model composition			
Non-hydrogen atoms	11138	11139	11139
Protein residues	1363	1363	1363
Water	10	11	11
Ligands	1 MG, 1 POV, 1 VN4, 4 NAG, 1 BMA	1 MG, 1 D39, 1 VN4, 4 NAG, 1 BMA	1 MG, 1 PIE, 1 VN4, 4 NAG, 1 BMA
B factors (Å^2, min/max/mean)	38.03/200.70/77.71	26.94/135.44/58.97	39.41/148.73/71.27
Protein	43.54/113.55/73.32	33.39/93.40/56.30	41.91/98.95/69.14
Ligand	37.79/63.26/51.22	30.62/43.60/36.24	44.02/54.97/50.12
Water			
R.m.s. deviations			
Bond lengths (Å)	0.003	0.003	0.003
Bond angles (°)	0.518	0.513	0.516
Validation			
MolProbity score	1.59	1.51	1.60
Clashscore	6.07	4.72	5.94
Ramachandran plot			
Favored (%)	96.22	96.15	96.0
Allowed (%)	3.78	3.85	4.0
Outliers (%)	0.0	0.0	0.0

Supplementary Table 1 - Data collection and refinement statistics

Primer Name	Sequence
ATP8B1 5´ (Forward)	TACCGAATTCTAGTATGACGGC
ATP8B1 3' (Reverse)	ACCGAGCTCTCAGCTGTC
ATP8B1-3Csite (Forward)	GAGGTGCTGTTCCAGGGCCCGAAGGCGGAGGAGCAGTGG
ATP8B1-3Csite (Reverse)	CGGGCCCTGGAACAGCACCTCCAACCGCTTGCGATGCTTC

Supplementary Table 2 – Primers used to insert a 3C protease site at position L1185 of ATP8B1

Supplementary Discussion

Is there a second magnesium ion in the AMPPCP binding pocket?

In our cryo-EM map of the ATP8B1-CDC50A complex trapped in the E1-ATP conformation with AMPPCP, a density could be observed at proximity of the beta-phosphate of the AMPPCP molecule (see picture below).

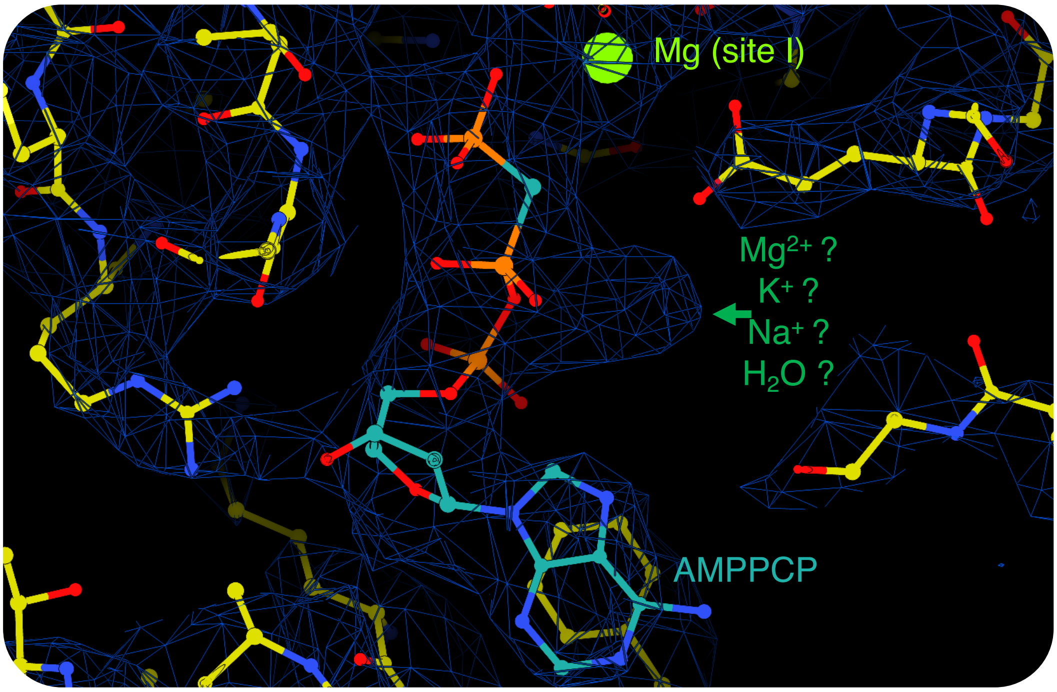

Close-up view of the electron density map (4.2σ) of AMPPCP and neighboring residues of ATP8B1, highlighting the presence of an unassigned extra density near the beta phosphate.

Previously, a second magnesium ion has been observed in P-type ATPase structrures trapped in a similar conformation using ADP and AlF_4[1,2]. However, to our knowledge, it has never been observed or modeled for P-type ATPase trapped with AMPPCP. In a recent study, Mateeva and colleagues employed molecular dynamics in combination with Quantum Mechanical/Molecular Mechanism (QM/MM) techniques to investigate the role of a second magnesium ion in this site[3]. According to their findings, a second magnesium ion, located at a position similar to our observed extra density, plays a critical role in the transfer of the gamma-phosphate of ATP to the catalytic aspartate of P-type ATPase.

Interestingly, during their MD simulation, Mateeva et al. also noticed that potassium ions (the only monovalent cation included in their study) can bind at the same position. To gain a better understanding of which element is responsible for the extra density observed in our map, we compared it with all available cryo-EM maps of P-type ATPase trapped with AMPPCP. We also examined the buffer composition of the sample used for grid freezing (see table below). Unfortunately, we could not find any clear correlation between the cations present in the samples and the presence of the extra density in the published cryo-EM maps. This suggests that different ions such as sodium (Na^+), potassium (K^+), or magnesium (Mg^{2+}) may partially occupy the site, in line with the weaker intensity of this density in all EM maps compared to the other magnesium site (site I). For consistency, we chose not to model any ion into this density.

Lastly, we did not model a second magnesium ion in our E1P-ADP model due to the uncertainty in our cryo-EM map in this region.

PDB	P-type ATPase	Last SEC buffer / + ligand before grid freezing	Cations?	EM density?	Reference
7KY8	Dnf2	20 mM HEPES, pH 7.4, **150 mM NaCl**, 0.003% LMNG, 0.0003% CHS and **5 mM $MgCl_2$** / 2 mM AMPPCP	Mg^{2+}, Na^+	YES	[4]Bai et al., 2020
6K7I	ATP8A2	20 mM HEPES-**Na**OH pH 7.5, **150 mM NaCl**, and 0.06% GDN / **5 mM $MgCl_2$** and 2 mM AMPPCP	Mg^{2+}, Na^+	? (low resolution)	[5]Hiraizumi et al., 2019
6K7J	ATP8A2	20 mM HEPES-**Na**OH pH 7.5, **150 mM NaCl**, and 0.06% GDN / **5 mM $MgCl_2$** and 2 mM AMPPCP	Mg^{2+}, Na^+	YES but AMPPCP is in a different conformation	[5]Hiraizumi et al., 2019
7BSP	ATP11C	20 mM MES-Tris pH 6.5, 1% glycerol, **50 mM NaCl**, **5 mM $MgCl_2$**, and 0.06% GDN / 2 mM AMPPCP	Mg^{2+}, Na^+	YES	[6]Nakanishi et al., 2020
7VPI	ATP13A2	20 mM Tris pH 8.0, **150 mM NaCl**, 1.5% GDN, and 5 mM DTT / **5 mM $MgCl_2$** and 2 mM AMPPCP	Mg^{2+}, Na^+	NO	[7]Tomita et al., 2021
6LCR	Dnf1	Not clear : (20 mM Tris-HCl pH 7.4, **150 mM NaCl**, **5 mM $MgCl_2$**, 1mM DTT) **?** / 1mM AMPPCP	**?** (Mg^{2+}, Na^+)	?	[8]He et al., 2022
7DSH	Dnf2	20 mM HEPES-**Na**OH pH 7.5, **150 mM NaCl**, **5 mM $MgCl_2$**, and 1 mM DTT / 2 mM AMPPCP	Mg^{2+}, Na^+	? (low resolution)	[9]Xu et al., 2022

7DSI	Dnf2	20 mM HEPES-**Na**OH pH 7.5, 150 mM NaCl, **5 mM $MgCl_2$**, and 1 mM DTT / 2 mM AMPPCP	Mg^{2+}, Na^{+}	YES	[9]Xu et al., 2022
7WHW	Dnf2	20 mM HEPES-**Na**OH pH 7.5, **150 mM NaCl**, **5 mM $MgCl_2$**, and 1 mM DTT / 2 mM AMPPCP	Mg^{2+}, Na^{+}	NO	[9]Xu et al., 2022
7OH7	Drs2	50 mM MOPS-Tris pH 7, **100 mM KCl**, 1 mM DTT, **5 mM $MgCl_2$** and 0.03 $mg.mL^{-1}$ LMNG / 2 mM AMPPCP	Mg^{2+}, K^{+}	NO	[10]Timcenko et al., 2021
7RD8	Neo1	20 mM HEPES pH 7.4, **150 mM NaCl**, **1 mM $MgCl_2$** and 0.025 $mg.mL^{-1}$ LMNG, 0.0025 $mg.mL^{-1}$ CHS, / **5mM $MgCl_2$**, and 2 mM AMPPCP	Mg^{2+}, Na^{+}	? (low resolution)	[11]Bai et al., 2021
6LLE	Serca2b	50 mM Hepes-**Na**OH pH 7.0, **100 mM KCl**, **1 mM $CaCl_2$**, **1 mM $MgCl_2$**, 1 mM DTT, and 0.1 $mg.mL^{-1}$ LMNG / 1 mM AMPPCP	Mg^{2+},Ca^{2+},K^{+}	YES	[12]Zhang et al., 2020
6LN5	Serca2b	50 mM Hepes-**Na**OH pH 7.0, **100 mM KCl**, **1 mM $CaCl_2$**, **1 mM $MgCl_2$**, 1 mM DTT, and 0.01% (w/v) LMNG / 1 mM AMPPCP	Na^{+} Mg^{2+},Ca^{2+},K^{+}	YES	[12]Zhang et al., 2020
6LN6	Serca2b	50 mM Hepes-**Na**OH pH 7.0, **100 mM KCl**, **1 mM $CaCl_2$**, **1 mM $MgCl_2$**, 1 mM DTT, and 0.01% (w/v) LMNG / 1 mM AMPPCP	Na^{+}, Mg^{2+},Ca^{2+},K^{+}	YES	[12]Zhang et al., 2020
6LN7	Serca2b	50 mM Hepes-**Na**OH pH 7.0, **100 mM KCl**, **1 mM $CaCl_2$**, **1 mM $MgCl_2$**, 1 mM DTT, and 0.01% (w/v) LMNG / 1 mM AMPPCP	Mg^{2+},Ca^{2+},K^{+}	YES	[12]Zhang et al., 2020
6XMQ	Spf1	20 mM Tris pH 7.5, **100 mM NaCl**, **1 mM EDTA**, **10 mM $MgCl_2$**, 1 mM DTT, 0.04% DDM, and 0.008% CHS / 2 mM of AMPPCP	Mg^{2+} ,Na^{+}	NO	[13]McKenna et al., 2020
7YAG	SPCA	50 mM MES pH 6.0, **100 mM NaCl**, **1 mM $CaCl_2$**, **1 mM $MgCl_2$**, 1 mM DTT, and 0.02% GDN / 1 mM AMPPCP	Mg^{2+}, Ca^{2+},Na^{+}	YES (but no Mg in site I)	[14]Chen et al., 2023
7YAH	SPCA	50 mM MES pH 6.0, **100 mM NaCl**, **1 mM $CaCl_2$**, **1 mM $MgCl_2$**, 1 mM DTT, and 0.02% GDN / 1 mM AMPPCP	Mg^{2+}, Ca^{2+},Na^{+}	YES (but no Mg in site I)	[14]Chen et al., 2023
7YAI	SPCA	50 mM MES pH 6.0, **100 mM NaCl**, **1 mM $CaCl_2$**, **1 mM $MgCl_2$**, 1 mM DTT, and 0.02% GDN / 1 mM AMPPCP	Mg^{2+}, Ca^{2+},Na^{+}	YES (but no Mg in site I)	[14]Chen et al., 2023
7YAJ	SPCA	50 mM MES pH 6.0, **100 mM NaCl**, **1 mM $MnCl_2$**, **1 mM $MgCl_2$**, 1 mM DTT, and 0.02% GDN / 1 mM AMPPCP	Mg^{2+}, Mn^{2+},Na^{+}	NO (but no Mg in site I)	[14]Chen et al., 2023
8D3W	Na/K Alpha 3	20 mM HEPES-**Na** pH 7.4, **150 mM NaCl** and 0.06% (w/v) GDN / 1 mM AMPPCP and **4 mM $MgCl_2$**	Mg^{2+}, Ca^{2+},Na^{+}	YES	[15]Nguyen et al., 2022
7LC3	KdpB	25 mM Tris pH 7.5, 10% glycerol, 1 mM TCEP, **100 mM NaCl**, and 0.15% n-decyl-β-maltoside / **100 mM K^{+}**, 5 mM AMPPCP	Na^{+}, K^{+}	NO	[16]Sweet et al., 2021

7NNL	KdpB	10 mM Tris-HCl pH 8, **10 mM $MgCl_2$, 10 mM NaCl** and 0.012% DDM / **50 mM KCl** and 5 mM AMPPCP	Mg^{2+}, Na^+, K^+	NO (low resolution)	[17]Silberberg et al., 2021
7NNP	KdpB	10 mM Tris-HCl pH 8, **10 mM $MgCl_2$, 10 mM NaCl** and 0.012% DDM / **100 mM RbCl**, 10 mM AMPPCP + residual ATP/ADP	Mg^{2+}, Na^+, Rb^+	YES? (low resolution)	[17]Silberberg et al., 2021

Supplementary References

1. Sørensen, T. L.-M., Møller, J. V. & Nissen, P. Phosphoryl Transfer and Calcium Ion Occlusion in the Calcium Pump. *Science* **304**, 1672–1675 (2004).
2. Kanai, R., Ogawa, H., Vilsen, B., Cornelius, F. & Toyoshima, C. Crystal structure of a Na+-bound Na+,K+-ATPase preceding the E1P state. *Nature* **502**, 201–206 (2013).
3. Mateeva, T., Klähn, M. & Rosta, E. Structural Dynamics and Catalytic Mechanism of ATP13A2 (PARK9) from Simulations. *J. Phys. Chem. B* **125**, 11835–11847 (2021).
4. Bai, L. *et al.* Transport mechanism of P4 ATPase phosphatidylcholine flippases. *eLife* **9**, e62163 (2020).
5. Hiraizumi, M., Yamashita, K., Nishizawa, T. & Nureki, O. Cryo-EM structures capture the transport cycle of the P4-ATPase flippase. *Science* **365**, 1149–1155 (2019).
6. Nakanishi, H. *et al.* Transport Cycle of Plasma Membrane Flippase ATP11C by Cryo-EM. *Cell Rep.* **32**, 108208 (2020).
7. Tomita, A. *et al.* Cryo-EM reveals mechanistic insights into lipid-facilitated polyamine export by human ATP13A2. *Mol. Cell* **81**, 4799-4809.e5 (2021).
8. He, Y., Xu, J., Wu, X. & Li, L. Structures of a P4-ATPase lipid flippase in lipid bilayers. *Protein Cell* **11**, 458–463 (2020).
9. Xu, J., He, Y., Wu, X. & Li, L. Conformational changes of a phosphatidylcholine flippase in lipid membranes. *Cell Rep.* **38**, 110518 (2022).
10. Timcenko, M. *et al.* Structural Basis of Substrate-Independent Phosphorylation in a P4-ATPase Lipid Flippase. *J. Mol. Biol.* **433**, 167062 (2021).
11. Bai, L. *et al.* Structural basis of the P4B ATPase lipid flippase activity. *Nat. Commun.* **12**, 5963 (2021).
12. Zhang, Y. *et al.* Cryo-EM structures of SERCA2b reveal the mechanism of regulation by the luminal extension tail. *Sci. Adv.* **6**, eabb0147 (2020).

13. McKenna, M. J. *et al.* The endoplasmic reticulum P5A-ATPase is a transmembrane helix dislocase. *Science* **369**, eabc5809 (2020).

14. Chen, Z. *et al.* Cryo-EM structures of human SPCA1a reveal the mechanism of Ca2+/Mn2+ transport into the Golgi apparatus. *Sci. Adv.* **9**, eadd9742 (2023).

15. Nguyen, P. T. *et al.* Structural basis for gating mechanism of the human sodium-potassium pump. *Nat. Commun.* **13**, 5293 (2022).

16. Sweet, M. E. *et al.* Structural basis for potassium transport in prokaryotes by KdpFABC. *Proc. Natl. Acad. Sci.* **118**, e2105195118 (2021).

17. Silberberg, J. M. *et al.* Deciphering ion transport and ATPase coupling in the intersubunit tunnel of KdpFABC. *Nat. Commun.* **12**, 5098 (2021).

Uncropped SDS-PAGE from Supplementary Figure 2

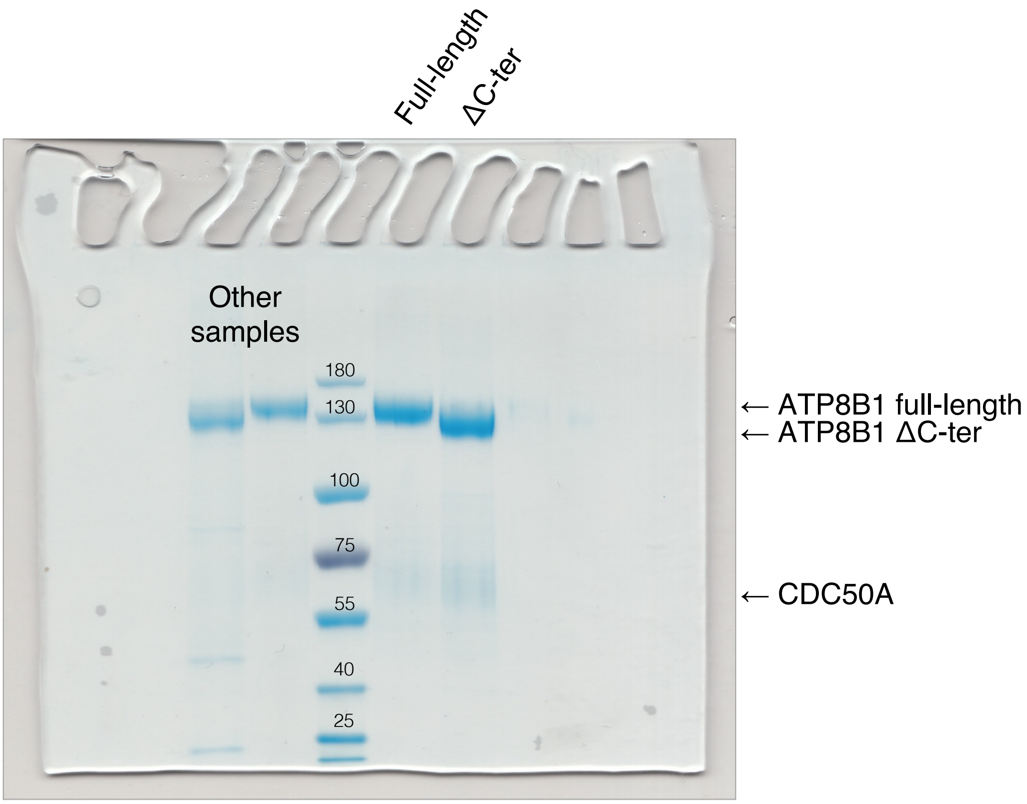

Supplement: Supplementary file 1 — Supplementary Information [file 41467_2023_42828_MOESM1_ESM.pdf]
