# Supplementary material for: Activation and substrate specificity of the human P4-ATPase ATP8B1
Source: Nat Commun. 2023 Nov 18;14:7492. doi: 10.1038/s41467-023-42828-9 (PMC10657443; doi:10.1038/s41467-023-42828-9)
Supplement: Supplementary file 3 — Description of Additional Supplementary Files [file 41467_2023_42828_MOESM3_ESM.pdf]

Description of Additional Supplementary Files

File name: Supplementary Movie 1

Description: Close up views of the lipid occlusion sites in the E2-Pi lipid occluded conformations in presence of phosphatidylcholine (PC), phosphatidylserine (PS), or phosphatidylinositol (PI) and their associated cryo-EM maps.
