# Supplementary material for: Activation and substrate specificity of the human P4-ATPase ATP8B1
Source: Nat Commun. 2023 Nov 18;14:7492. doi: 10.1038/s41467-023-42828-9 (PMC10657443; doi:10.1038/s41467-023-42828-9)
Supplement: Supplementary file 5 — Reporting Summary [file 41467_2023_42828_MOESM5_ESM.pdf]

nature portfolio

Corresponding author(s): Thibaud Dieudonné
Poul Nissen

Last updated by author(s): Oct 6, 2023

Reporting Summary

Nature Portfolio wishes to improve the reproducibility of the work that we publish. This form provides structure for consistency and transparency in reporting. For further information on Nature Portfolio policies, see our Editorial Policies and the Editorial Policy Checklist.

Statistics

For all statistical analyses, confirm that the following items are present in the figure legend, table legend, main text, or Methods section.

n/a	Confirmed	
☐	☒	The exact sample size (*n*) for each experimental group/condition, given as a discrete number and unit of measurement
☐	☒	A statement on whether measurements were taken from distinct samples or whether the same sample was measured repeatedly
☒	☐	The statistical test(s) used AND whether they are one- or two-sided<br>*Only common tests should be described solely by name; describe more complex techniques in the Methods section.*
☒	☐	A description of all covariates tested
☒	☐	A description of any assumptions or corrections, such as tests of normality and adjustment for multiple comparisons
☐	☒	A full description of the statistical parameters including central tendency (e.g. means) or other basic estimates (e.g. regression coefficient) AND variation (e.g. standard deviation) or associated estimates of uncertainty (e.g. confidence intervals)
☒	☐	For null hypothesis testing, the test statistic (e.g. *F*, *t*, *r*) with confidence intervals, effect sizes, degrees of freedom and *P* value noted<br>*Give P values as exact values whenever suitable.*
☒	☐	For Bayesian analysis, information on the choice of priors and Markov chain Monte Carlo settings
☒	☐	For hierarchical and complex designs, identification of the appropriate level for tests and full reporting of outcomes
☒	☐	Estimates of effect sizes (e.g. Cohen's *d*, Pearson's *r*), indicating how they were calculated

Our web collection on statistics for biologists contains articles on many of the points above.

Software and code

Policy information about availability of computer code

Data collection	EPU (FEI/THermoFisher) was used for automated collection of cryo-EM data. For ATPase activity measurements, NADH absorbance decay was measured in a SpectraMax®i3 plate reader with the SoftMax® Pro Software. AlphaFold2, MODELLER v10.1, CHARMM-GUI Membrane Builder web tool and CHARMM-GUI Input Generator were used for system preparation and setup. GROMACS v2020/3 was used for all simulations All, but EPU and the SoftMax® Pro and are open source.
Data analysis	Cryo-EM data was analyzed through cryoSPARC v3 package. Modelling and model validation was performed using COOT 0.9 and Phenix 1.19-4092 was used with the Molprobity package. For ATPase activity measurements, NADH absorbance data were analysis in Microscoft Excel. For simulation, GROMACS v2019/4 was used for processing of the trajectories and the clustering analysis. The remaining analysis was performed in python v3.7 using the libraries MDAnalysis v2.0.0-dev0 and GetContacts (downloaded 02/2021). Data visualisation was performed using python v3.7 and ChimeraX v1.4. All, but cryoSPARC and Microsoft Excel are open source.<br>All, but cryoSPARC and Microsoft Excel are open source.

For manuscripts utilizing custom algorithms or software that are central to the research but not yet described in published literature, software must be made available to editors and reviewers. We strongly encourage code deposition in a community repository (e.g. GitHub). See the Nature Portfolio guidelines for submitting code & software for further information.

Data

Policy information about availability of data

All manuscripts must include a data availability statement. This statement should provide the following information, where applicable:

- Accession codes, unique identifiers, or web links for publicly available datasets
- A description of any restrictions on data availability
- For clinical datasets or third party data, please ensure that the statement adheres to our policy

The protein sequences of ATP8B1 and CDC50A corresponds to the Uniprot references O43520; A1152T natural variant and Q9NV96, respectively. The ATP8B1-CDC50A models were built using the previously published structure of the complex (PDB: 7PY4) as template. Cryo-EM density maps have been deposited in the Electron Microscopy Data Bank under the accession codes EMD-17256 (E1-ATP), EMD-17257 (E1P-ADP), EMD-17258 (E1P), EMD-17259 (E2Pautoinhibited "closed"), EMD-17260 (E2Pautoinhibited "active"), EMD-17261 (E2Pactive), EMD-17262 (E2?Pi (PS)), EMD-17262 (E2?Pi (PC)) and EMD-17263 (E2?Pi (PI)). Atomic coordinates have been deposited in the Protein Data Bank under IDs 8OX4 (E1-ATP), 8OX5 (E1P-ADP), 8OX6 (E1P), 8OX7 (E2Pautoinhibited "closed"), 8OX8 (E2Pautoinhibited "active"), 8OX9 (E2Pactive), 8OXA (E2?Pi (PS)), 8OXA (E2?Pi (PC)) and 8OXC (E2?Pi (PI)). MD data and analysis have been deposited to ERDA and are available from : http://doi.org/10.17894/ucph.44e191c6-97ad-43ef-944f-be2ad95329fd. The data underlying Figs. 4a, 6b, and 7c are provided as a Source Data file. Source data are provided with this paper.

Research involving human participants, their data, or biological material

Policy information about studies with human participants or human data. See also policy information about sex, gender (identity/presentation), and sexual orientation and race, ethnicity and racism.

Reporting on sex and gender	Not relevant as there are no human research participant
Reporting on race, ethnicity, or other socially relevant groupings	Not relevant as there are no human research participant
Population characteristics	Not relevant as there are no human research participant
Recruitment	Not relevant as there are no human research participant
Ethics oversight	Not relevant as there are no human research participant

Note that full information on the approval of the study protocol must also be provided in the manuscript.

Field-specific reporting

Please select the one below that is the best fit for your research. If you are not sure, read the appropriate sections before making your selection.

☒ Life sciences ☐ Behavioural & social sciences ☐ Ecological, evolutionary & environmental sciences

For a reference copy of the document with all sections, see nature.com/documents/nr-reporting-summary-flat.pdf

Life sciences study design

All studies must disclose on these points even when the disclosure is negative.

Sample size	The sample size was not determined
Data exclusions	No data were excluded from the analysis
Replication	The purification of the ATP8B1-CDC50A complex has been repeated at least 4 times for this study. For cryo-EM data, multiple grids were prepared and the data for each data set has been collected on one selected grid. For ATPase activity measurement, all experiments have been repeated at least three times with the same purified complex as stated in the text. For MD simulations, the simulation were repeated five times.
Randomization	Not relevant as there are no experimental groups
Blinding	Not relevant as there are no experimental groups

Behavioural & social sciences study design

All studies must disclose on these points even when the disclosure is negative.

Study description	Not relevant to our study
Research sample	Not relevant to our study
Sampling strategy	Not relevant to our study
Data collection	Not relevant to our study
Timing	Not relevant to our study
Data exclusions	Not relevant to our study
Non-participation	Not relevant to our study
Randomization	Not relevant to our study

Ecological, evolutionary & environmental sciences study design

All studies must disclose on these points even when the disclosure is negative.

Study description	Not relevant to our study
Research sample	Not relevant to our study
Sampling strategy	Not relevant to our study
Data collection	Not relevant to our study
Timing and spatial scale	Not relevant to our study
Data exclusions	Not relevant to our study
Reproducibility	Not relevant to our study
Randomization	Not relevant to our study
Blinding	Not relevant to our study

Did the study involve field work? ☐ Yes ☒ No

Field work, collection and transport

Field conditions	Not relevant to our study
Location	Not relevant to our study
Access & import/export	Not relevant to our study
Disturbance	Not relevant to our study

Reporting for specific materials, systems and methods

We require information from authors about some types of materials, experimental systems and methods used in many studies. Here, indicate whether each material, system or method listed is relevant to your study. If you are not sure if a list item applies to your research, read the appropriate section before selecting a response.

Materials & experimental systems

n/a	Involved in the study
☒	☐ Antibodies
☐	☒ Eukaryotic cell lines
☒	☐ Palaeontology and archaeology
☒	☐ Animals and other organisms
☒	☐ Clinical data
☒	☐ Dual use research of concern
☒	☐ Plants

Methods

n/a	Involved in the study
☒	☐ ChIP-seq
☒	☐ Flow cytometry
☒	☐ MRI-based neuroimaging

Antibodies

Antibodies used	Not relevant to our study
Validation	Not relevant to our study

Eukaryotic cell lines

Policy information about cell lines and Sex and Gender in Research

Cell line source(s)	Saccharomyces cerevisiae W303.1b/Δpep4
Authentication	Not Authenticated
Mycoplasma contamination	Not applicable
Commonly misidentified lines (See ICLAC register)	Not applicable

Palaeontology and Archaeology

Specimen provenance	Not applicable
Specimen deposition	Not applicable
Dating methods	Not applicable

☐ Tick this box to confirm that the raw and calibrated dates are available in the paper or in Supplementary Information.

Ethics oversight	Not applicable

Note that full information on the approval of the study protocol must also be provided in the manuscript.

Animals and other research organisms

Policy information about studies involving animals; ARRIVE guidelines recommended for reporting animal research, and Sex and Gender in Research

Laboratory animals	Not applicable
Wild animals	Not applicable
Reporting on sex	Not applicable
Field-collected samples	Not applicable
Ethics oversight	Not applicable

Note that full information on the approval of the study protocol must also be provided in the manuscript.

Clinical data

Policy information about clinical studies

All manuscripts should comply with the ICMJE guidelines for publication of clinical research and a completed CONSORT checklist must be included with all submissions.

Clinical trial registration	Not applicable
Study protocol	Not applicable
Data collection	Not applicable
Outcomes	Not applicable

Dual use research of concern

Policy information about dual use research of concern

Hazards

Could the accidental, deliberate or reckless misuse of agents or technologies generated in the work, or the application of information presented in the manuscript, pose a threat to:

No	Yes	
☒	☐	Public health
☒	☐	National security
☒	☐	Crops and/or livestock
☒	☐	Ecosystems
☒	☐	Any other significant area

Experiments of concern

Does the work involve any of these experiments of concern:

No	Yes	
☒	☐	Demonstrate how to render a vaccine ineffective
☒	☐	Confer resistance to therapeutically useful antibiotics or antiviral agents
☒	☐	Enhance the virulence of a pathogen or render a nonpathogen virulent
☒	☐	Increase transmissibility of a pathogen
☒	☐	Alter the host range of a pathogen
☒	☐	Enable evasion of diagnostic/detection modalities
☒	☐	Enable the weaponization of a biological agent or toxin
☒	☐	Any other potentially harmful combination of experiments and agents

Plants

Seed stocks	Not applicable
Novel plant genotypes	Not applicable
Authentication	Not applicable

ChIP-seq

Data deposition

☐ Confirm that both raw and final processed data have been deposited in a public database such as GEO.

☐ Confirm that you have deposited or provided access to graph files (e.g. BED files) for the called peaks.

Data access links<br>*May remain private before publication.*	Not applicable
Files in database submission	Not applicable
Genome browser session<br>(e.g. UCSC)	Not applicable

Methodology

Replicates	Not applicable
Sequencing depth	Not applicable
Antibodies	Not applicable
Peak calling parameters	Not applicable
Data quality	Not applicable
Software	Not applicable

Flow Cytometry

Plots

Confirm that:

☐ The axis labels state the marker and fluorochrome used (e.g. CD4-FITC).

☐ The axis scales are clearly visible. Include numbers along axes only for bottom left plot of group (a 'group' is an analysis of identical markers).

☐ All plots are contour plots with outliers or pseudocolor plots.

☐ A numerical value for number of cells or percentage (with statistics) is provided.

Methodology

Sample preparation	Not applicable
Instrument	Not applicable
Software	Not applicable
Cell population abundance	Not applicable
Gating strategy	Not applicable

☐ Tick this box to confirm that a figure exemplifying the gating strategy is provided in the Supplementary Information.

Magnetic resonance imaging

Experimental design

Design type	Not applicable
Design specifications	Not applicable
Behavioral performance measures	Not applicable

Acquisition

Imaging type(s)	Not applicable
Field strength	Not applicable
Sequence & imaging parameters	Not applicable
Area of acquisition	Not applicable

Diffusion MRI ☐ Used ☒ Not used

Preprocessing

Preprocessing software	Not applicable
Normalization	Not applicable
Normalization template	Not applicable
Noise and artifact removal	Not applicable
Volume censoring	Not applicable

Statistical modeling & inference

Model type and settings	Not applicable
Effect(s) tested	Not applicable

Specify type of analysis: ☐ Whole brain ☐ ROI-based ☐ Both

Statistic type for inference (See Eklund et al. 2016)	Not applicable
Correction	Not applicable

Models & analysis

n/a	Involved in the study
☐	☒ Functional and/or effective connectivity
☐	☐ Graph analysis
☐	☐ Multivariate modeling or predictive analysis

Functional and/or effective connectivity	Not applicable
Graph analysis	Not applicable
Multivariate modeling and predictive analysis	Not applicable
